# Discovering structure–property relationships for the phonon band structures of hydrocarbon-based organic semiconductor crystals: the instructive case of acenes†

**DOI:** 10.1039/d1tc04708f

**Published:** 2021-10-15

**Authors:** Tomas Kamencek, Egbert Zojer

**Affiliations:** Institute of Solid State Physics, Graz University of Technology NAWI Graz Petersgasse 16 8010 Graz Austria egbert.zojer@tugraz.at; Institute of Physical and Theoretical Chemistry, Graz University of Technology NAWI Graz Stremayrgasse 9 8010 Graz Austria

## Abstract

By studying the low-frequency phonon bands of a series of crystalline acenes, this article lays the foundation for the development of structure–property relationships for phonons in organic semiconductors. Combining state-of-the art quantum–mechanical simulations with simple classical models, we explain how and why phonon frequencies and group velocities do or do not change when varying the molecular and crystal structures of the materials.

## Abstract 2

A systematic analysis of the phonon bands calculated using dispersion-corrected density functional theory for crystalline acenes reveals analogies between the phonons and classical oscillator models. Based on these, the evolution of the materials’ properties can be rationalised.

## Graphical abstract

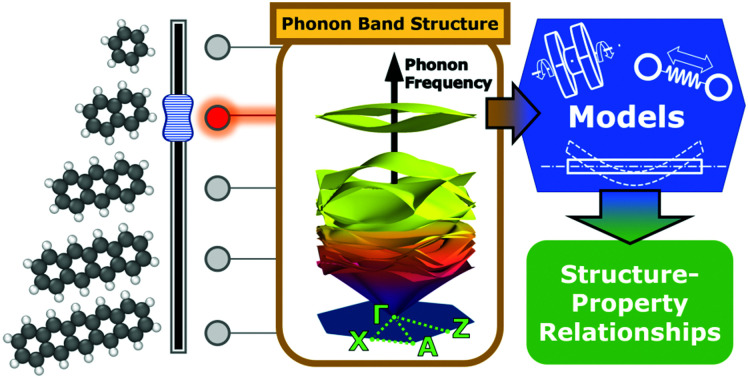

## Introduction

Organic semiconductors (OSCs) are a scientifically and technologically appealing class of materials, which allow the realisation of cheap electronic systems with specifically tuned properties, and which exploit the incredibly versatile toolbox of organic chemistry.^1,2^ Devices built from such materials include organic light-emitting diodes,^3^ solar cells,^4,5^ or thin-film transistors.^6^ Many of the physical properties of OSCs are crucially influenced by phonons, which are the quasiparticles of lattice vibrations. They impact thermal properties, such as the heat capacity or free energy, including the phonon contribution to a material's entropy. These quantities are crucial for assessing the relative thermodynamic stability of polymorphs,^7–10^ which are often energetically very close.^11,12^ Moreover, essentially all practically relevant transport properties of an OSC crystal are determined by phonons. This applies to the crystal's thermal conductivity,^13–15^ its ability to convert heat to electric energy (*i.e.*, its thermoelectric performance),^16–18^ and, *via* the particularly strong electron–phonon coupling, its charge-carrier mobility.^19–31^ For the latter, certain killer phonon modes have already been identified in the literature.^29^ Due to their particularly strong coupling with parameters relevant to charge transport (like the intermolecular transfer integrals) they have been identified as the key reason for charge-carrier mobilities in most OSC materials being much lower than expected.^29^

Consequently, a precise understanding of phonons in OSCs is key to understanding the above-mentioned properties. All harmonic characteristics of phonons are encoded in the respective phonon band structures. Of particular relevance in this context is the development of a clear picture of how phonon properties and band structures are affected by aspects like the molecular packing and the structure, length, and shape of the molecules that constitute the OSC. Unfortunately, so far, comprehensive structure–property relationships for phonons in OSCs are largely missing.

In order to provide a starting point for their development, in the following, we present a detailed discussion of phonons of a series of acenes, which are arguably one of the most central families of organic semiconducting materials. Unfortunately, an experimental determination of phonon band structures is extremely challenging for these materials, as for the commonly employed neutron scattering experiments, one requires comparatively large single crystals, which also need to be fully deuterated to reduce the fraction of incoherent scattering events. Consequently, there are only very few, scattered, examples of crystals consisting of conjugated molecules for which phonon band structures have been measured. These comprise, for example, deuterated naphthalene^32,33^ and anthracene,^34^ for which the dependence of the bands on temperature,^32,35–37^ pressure,^38^ and on the anharmonic nature of the phonons^39^ have been determined. Thus, for our discussion we will rely on suitably benchmarked dispersion-corrected density-functional-theory (DFT) calculations. These have the additional benefit of providing direct access to the atomistic motions that describe specific phonon modes. The methodology employed here can be used with great confidence, bearing in mind that it shows excellent agreement with the experimentally determined phonon bands of the above-mentioned deuterated naphthalene.^40–42^ Most challenging for the simulations is the accurate description of the intermolecular phonon region (up to ∼6 THz ≈ 200 cm^−1^), as it is strongly impacted by intermolecular van der Waals (vdW) interactions that are not properly accounted for in semi-local DFT. Therefore, we benchmarked a variety of vdW corrections against available experimental Raman data^43^ and phonon band structures^40^ for OSCs in that region. For the corrections applied here, RMS deviations between measured and simulated phonon frequencies on the order of ∼0.13 THz (∼4.3 cm^−1^) have been obtained.^40,43^ Important ingredients for phonon calculations are also the crystal structures of the studied materials, where we started from (existing) OSC crystal structures documented in the Cambridge Structural Database (see below).^44^

In the following we provide a systematic discussion of the phonon properties of a series of acenes from benzene to pentacene. This involves characterisation of the various phonon modes, especially with respect to their evolution with molecular length. Also here, the primary focus will be on the low-frequency region (≤7 THz ≈ 230 cm^−1^), which comprises all intermolecular phonons and the lowest intramolecular bands, and which is particularly relevant for transport processes, as the corresponding modes are thermally occupied.^40^ The observed modes comprise molecular translations (the killer phonon modes) and rotations as well as molecular bending and torsional motions. In addition, we analyse the group velocities of the phonons, concentrating primarily on the acoustic bands (*i.e.*, the sound velocities in the materials, which can reach several kilometres per second), and finally we will discuss the impact of the phonon properties on the temperature dependence of the heat capacity as an illustrative example of a thermodynamic property. In the spirit of structure–property relationships, our results show that the evolution with molecular length differs considerably between different phonon modes (*i.e.*, different types of vibrations). In the majority of cases the evolution can, however, still be explained by simple classical models building on the changes in molecular masses, moments of inertia, unit-cell sizes, and estimated intermolecular force constants. These “molecule-based” trends are then modified by the differences in the packing motifs of the different acenes.

## Methods

All phonon band structures were calculated using the Phonopy package^45^ (version 1.13.2) based on atomic forces calculated within density functional theory^46,47^ (DFT) employing the Vienna *ab initio* simulation package^48–51^ (VASP; version 5.4.1). DFT calculations were carried out using the PBE functional,^52^ the standard pseudopotentials^53^ for the projector-augmented wave method,^54^ and a plane wave energy cutoff of 900 eV. Given its outstanding performance compared with other *a posteriori* van der Waals corrections for calculating the properties of low-frequency phonons in organic semiconductors,^40–43^ we employed the D3 van der Waals correction^55^ with Becke–Johnson damping^56^ (D3–BJ).

Prior to phonon calculations, the geometries of the various systems originally obtained from the Cambridge Structural Database^44^ were optimised in terms of their atomic positions and lattice parameters based on the Rose–Vinet equation of state^57^ to avoid Pulay stresses,^58^ as described in more detail in ref. 40. Phonon band structures were calculated for suitably converged supercells and using a finite displacement approach (displacement distance of 0.01 Å). Further details regarding the employed simulation parameters, the meshes for the discrete ***q***-point sampling and the calculation of densities of states per group velocity and frequency can be found in Section S4 (ESI†). The produced output and the required input to reproduce our results can be accessed *via* the NOMAD database.^59^

The phonon displacement patterns and the animations in the ESI† were generated by visualising the geometries displaced along the normal mode coordinates using the Ovito software (version 3.3.1).^60^ The structural view of pentacene in Fig. 3(c) was plotted using Vesta (version 3.5.5).^61^

## Results and discussion

### Structures of the studied systems

All acenes discussed below crystallize in a herringbone pattern with two molecules per unit cell (*Z* = 2). The molecules are arranged in planes parallel to the *a*_1_ and *a*_2_ axes. The choice of the order is such that *a*_1_ (*a*_2_) denotes the longer (shorter) distance of symmetry equivalent molecules, while the *a*_3_ axis is roughly parallel to the long molecular axis (for more details see Section S3, ESI†). Benzene^62^ (1A), naphthalene^63^ (2A) and anthracene^64^ (3A) crystallize in monoclinic lattices, while crystals of tetracene^65^ (4A) and the two considered pentacene polymorphs^66,67^ (5A and 5A-II) belong to the triclinic crystal class. In passing we also note that an orthorhombic benzene polymorph^68^ (1A-o) exists, which is thermodynamically more stable at low temperatures (and zero pressure).^69,70^ Due to its fundamentally different structure, its properties are described only in Section S9 (ESI†). Moreover, although at room temperature polymorph 5A-II is more stable than 5A, we will primarily focus on 5A as again its structure is more consistent with the structures of the shorter acenes, as explained in Section S3 (ESI†). The latter also contains an in-depth comparison of the lattice parameters and the arrangements of the two molecules per unit cell in the different acenes. This comparison shows that there are two groups of systems: in 1A, 2A, and 3A, the two molecules per unit cell tilt symmetrically into directions above and below the *a*_1_ axis (space group number 14); conversely, in the longer acenes, both molecules tilt essentially in the same direction such that their glide planes and screw axes are lost (space group number 2) and the surroundings of the two molecules per unit cell are no longer symmetry-equivalent. 1A also stands out somewhat within the first group of systems due to the significantly increased angle between the short molecular axes of the two molecules per unit cell, and due to particularly large normal distances between the planes of equivalent molecules in adjacent layers (for more details see Section S3 (ESI†) and the animations of the phonon modes). All these structural differences are insofar relevant, as they affect the intermolecular interactions, whose impact is superimposed on trends in the phonon characteristics that follow from purely molecular properties, such as molecular lengths, masses, and moments of inertia.

### Low-frequency phonon band structures

**Fig. 1:**
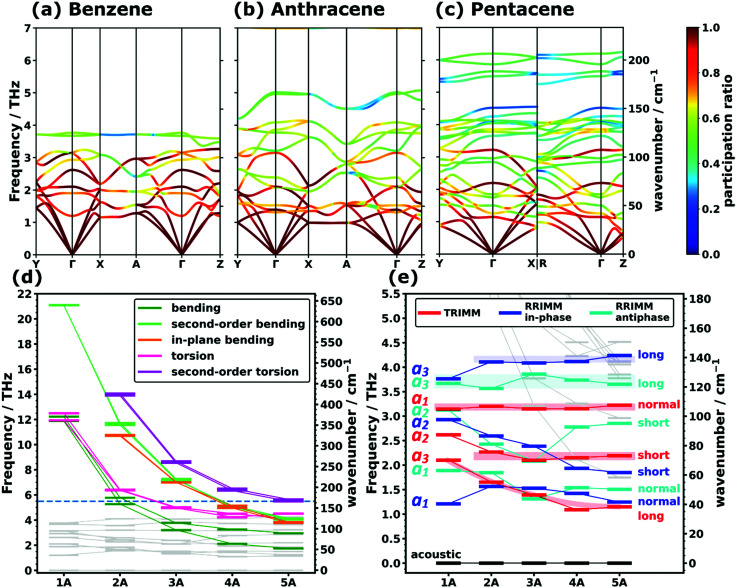
PBE/D3–BJ-calculated phonon band structures coloured according to the mode participation ratios of (a) the monoclinic polymorph of benzene, (b) anthracene, and (c) polymorph I of pentacene. For a description of the corresponding high-symmetry paths in the first Brillouin zones and the mathematical definition of the participation ratio see Sections S6 and S4.5 (ESI†). Panels (d) and (e) show the evolution of intramolecular (highlighted in (d)) and intermolecular (highlighted in (e)) frequencies at the *Γ*-point as a function of the molecular length. The frequencies are displayed as short horizontal bars, with the connecting lines serving as a guide to the eye. The horizontal dashed line in (d) marks the frequency interval shown in (e). The types of translational rigid intermolecular modes (TRIMMs) and rotational rigid intermolecular modes (RRIMs) are labelled with the lattice vectors ***a***_*i*_ and the molecular directions (long axis, short axis and the axis normal to the π-plane) along/around which the vibrations primarily occur. Animations of the displayed modes can be found in the ESI.† The shaded areas act as a guide to the eye and emphasise the observed trends for the selected intermolecular modes.

In view of the above discussion, we will focus on the low-frequency region up to ∼7 THz comprising those phonons that are most relevant, *e.g.*, for heat and charge transport. For a more quantitative discussion see ref. 40. Exemplary phonon band structures for monoclinic benzene,^62^ anthracene,^64^ as well as for the triclinic polymorph I of pentacene^66^ are shown in Fig. 1(a–c). The phonon band structures of naphthalene,^63^ tetracene,^65^ the thermodynamically more stable^69,70^ orthorhombic benzene (with four molecules in the unit cell),^68^ and of polymorph II of pentacene^67^ are given in Section S6 (ESI†). Up to ∼4 THz, the majority of the bands represent intermolecular phonons, which are characterized by motions of the molecules as more or less rigid objects. Of the twelve intermolecular bands (six per molecule in the unit cell), there are three acoustic and nine optical branches, where the latter comprise three translational and six rotational modes. These intermolecular bands are typically characterized by comparably large so-called participation ratios (PRs; see the colouring of the bands in Fig. 1(a–c)). The PRs range between 1 (corresponding to a mode in which each of the *N* atoms in the unit cell moves with the same amplitude) and *N*^−1^ (corresponding to a mode in which only one atom in the unit cell moves) and they describe the degree to which all atoms of the unit cell participate in the respective motion. Thus, they are a measure of the degree of delocalization of a mode (a rigorous mathematical definition of the PRs is given in Section S4.5, ESI†).^71–74^ As these intermolecular modes involve the motion of rather large masses (*i.e.*, entire molecules), which are bonded by relatively weak van der Waals forces, it is not surprising that they dominate the phonon properties at low frequencies. Nevertheless, for longer acenes, one still observes an increasing number of bands in the displayed spectral range despite the constant number of intermolecular phonon bands. This is a result of the increasing number of intramolecular modes that are shifted into the low-frequency region with increasing molecular length due to the associated higher molecular flexibility. This finding is in agreement with experimental observations for anthracene in the literature.^34^

To be able to analyse the situation more systematically, Fig. 1(d) and (e) show the evolutions of the *Γ*-point frequencies of all low-frequency modes. Panel (d), which is plotted over a wider frequency range, highlights the different intramolecular modes, while panel (e) focuses on the intermolecular ones.

### Intramolecular modes

**Fig. 2:**
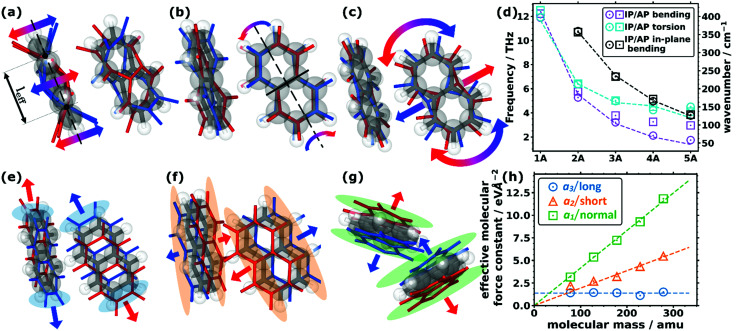
Displacement patters of the lowest intramolecular (a) bending, (b) torsional, and (c) in-plane bending modes for the two molecules in the unit cell of naphthalene. The equilibrium positions of the molecules (C grey, H white) are shown as grey and white sticks and semi-transparent balls, while the displaced positions are shown as coloured sticks (red and blue for the positive and negative amplitudes, respectively). The arrows indicate the most important motions. The effective bending length, *l*_eff_, is indicated in (a) as the distance between the two nodes of the bending amplitude. The nodal plane of the torsional mode in (b) is indicated by the solid black line. (d) Decrease in the frequency of the (in-plane) bending and torsional modes as a function of the size of the molecule for the five studied acenes showing the in-phase (IP) and antiphase (AP) frequencies. The models (corresponding to the dashed lines) are explained in more detail in Section S7 (ESI†). (e–g) Displacement patterns of the translational rigid intermolecular modes (TRIMMs) of naphthalene (e) along the lattice vector ***a***_3_, (f) along ***a***_2_, and (g) along ***a***_1_. The coloured semi-transparent regions emphasise the parts in space where the most pronounced changes of the (geometric) molecular overlap are observed. (h) Calculated effective intermolecular mode force constant values for the three TRIMMS in the studied molecular crystals as a function of the molecular mass. The dashed lines correspond to fitted linear functions through the origin for the TRIMMS along ***a***_3_ (considering all systems for that fit) and ***a***_2_ (omitting benzene for the fit for reasons explained in the main text). For the TRIMM along ***a***_1_, a constant was fitted.

As far as the intramolecular modes are concerned, in the low-frequency region, one is not dealing with stretching or bending motions of specific bonds, but rather with distortions of the entire molecular backbone. This is again a consequence of the larger associated masses and smaller associated force constants. As quantified below and shown in Fig. 1(d), the energies of these modes drop significantly for longer acenes. Consequently, for benzene there is still a pronounced band gap between intra- and intermolecular modes, which amounts to ∼8.1 THz. However, already for naphthalene the intramolecular bands approach the intermolecular region and for longer acenes the intra- and intermolecular bands overlap. To be more specific, the intramolecular modes of up to ∼14 THz comprise out-of-plane and in-plane backbone bending as well as backbone torsion vibrations. As there are two molecules per unit cell, each intramolecular vibrational mode results in two bands, with one corresponding to an in-phase (IP) and the other to an antiphase (AP) motion of the molecules. The ensuing displacements for the case of naphthalene are illustrated in Fig. 2(a) (with animations in the ESI†). In passing we note that for the in-plane bending and backbone torsion vibrations, the in-phase and antiphase vibrations are very close in energy (*i.e.*, their difference is sometimes rather poorly resolved in the plot in Fig. 1(d)).

To explain the shift of the bending modes with acene lengths, we resort to the model of a classical bending beam, whose eigenfrequency is inversely proportional to the square of its length (which, as argued in Section S7.1 (ESI†), is associated with the distance between the nodes of its fundamental vibration, *l*_eff_). Taking naphthalene as a reference, this yields the following relation for the frequency ratios of the corresponding modes in acenes:1
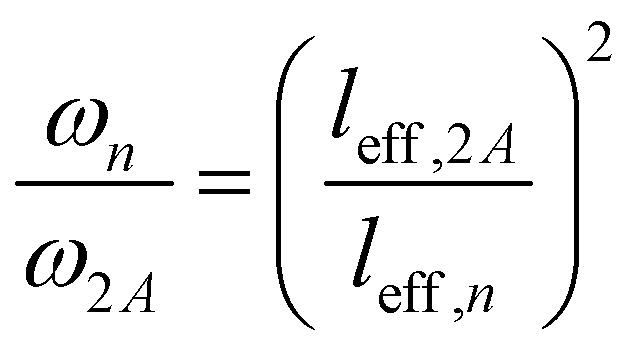


The evolution of *ω*_*n*_ obtained from eqn (1) perfectly matches the trend of the band positions in the actual band structures, as shown in Fig. 2(d). An analogous situation also applies to the in-plane bending modes, although with a slightly altered effective length and a higher stiffness (as motivated in Section S7.1, ESI†). Interestingly, the model from eqn (1) primarily follows the evolution of the in-phase modes. This is most pronounced for the backbone bending case, where an increase in the difference between the in-phase and antiphase energies with molecular length is clearly resolved. The differences can be understood as a consequence of the significantly increased intermolecular interactions for antiphase vibrations, which increases their apparent stiffness and, thus, the associated frequencies. This effect becomes particularly relevant for the larger displacement amplitudes of the central regions of the molecules.

A similar classical model can also be used to explain the decrease of torsional frequencies with *n*, as shown also in Fig. 2(d). The crucial factors here are (i) the torsional moment of inertia and (ii) the torsional stiffness. Both quantities not only depend on the molecular lengths, but also on whether the nodal plane of the torsional vibration at the centre of the molecule cuts through a phenylene ring or lies at the interface between two rings (as detailed in Section S7.2, ESI†). The latter gives rise to the odd–even effects observed in the evolution of the model data and the actual *Γ*-point frequencies, which are visible in Fig. 2(d).

Note that all the intramolecular modes discussed above show significant shifts to higher frequencies in the crystalline systems compared with the equivalent eigenmodes in the isolated molecules. This is the consequence of the increase of the associated force constants due to the intermolecular interactions in the crystals (a more detailed discussion of these shifts can be found in Section S11, ESI†).

### Intermolecular modes

Concerning the modes characterized primarily by motions of rigid molecules, several different cases need to be distinguished: the in-phase translational oscillations of the two molecules in the unit cell form the acoustic bands, which in all acenes display an almost perfectly linear dispersion over a comparably large frequency range (see Fig. 1(a–c)). Moreover, their band widths are relatively similar for different directions in reciprocal space. A more in-depth discussion of band dispersions and differences between transverse and longitudinal modes will be provided below, when analysing the corresponding group velocities. At this stage we would, however, like to comment on the widths of the acoustic bands. Strictly speaking, they decrease with the lengths of the acenes, but with the aid of the participation ratios (*i.e.*, by following the nearly linear band fragments with particularly high PRs) one can conclude that this is primarily a consequence of avoided band crossings, which occur as a consequence of phonon hybridisations involving other intermolecular bands belonging to the same irreducible symmetry representation. This effect is particularly pronounced for the triclinic crystals of tetracene and pentacene. Avoided crossings cause the opening of small band gaps, whose widths depend on the hybridisation strength. They are unfavourable for heat transport because of the (partial) loss of more strongly dispersing bands.^75–77^

Antiphase translational modes with the two molecules moving in opposite directions form optical bands, which are also characterized by particularly high degrees of delocalisation (PR ≈ 1). Three such bands exist in the systems considered here, and the evolution of their *Γ*-point frequencies is shown by the red symbols in Fig. 1(d). These **t**ranslational **r**igid **i**nter**m**olecular **m**odes (which we will refer to as TRIMMs) have a pronounced impact on the materials' charge-transport properties, as they comprise motions that result in massive variations of intermolecular electronic couplings.^19^ Thus, they are a major source of dynamic disorder and have been associated with killer phonon modes.^29^ Consequently, pushing them to higher frequencies to reduce the thermal occupation of these modes is expected to improve the carrier mobilities.^22,24–29^ Extending the conjugated backbones apparently does not have this effect, as illustrated in Fig. 1(e).

Interestingly, the evolution of each of the three TRIMMs with chain length appears to be fundamentally different with either decreasing or constant energy values. To understand this, one has to analyse the corresponding real space molecular displacements, which are shown in Fig. 2(e–g) (for animations see ESI†). It turns out that in all systems, the lowest frequency TRIMM corresponds to a translational vibration of the molecules along the ***a***_3_ axis, which is largely parallel to the long molecular axes (***a***_3_/long; see Fig. 2(e)). For naphthalene and longer acenes, this translation is combined with a minor bending of the molecular backbones. The next higher TRIMM is dominated by an antiphase translation of the two molecules in the ***a***_2_ direction, which is the lattice parameter at the smallest angle to the short molecular axes (***a***_2_/short; see Fig. 2(f)). Typically, these vibrations involve hardly any distortion of the molecules; only in tetracene a particularly strong hybridisation with the in-phase bending mode occurs due to the near degeneracy of that mode and the TRIMM. The highest-energy mode corresponds to an antiphase translation in the ***a***_1_ direction, which primarily causes a periodic variation of the distances normal to the molecular π-planes (***a***_1_/normal; see Fig. 2(g)). Here, for anthracene and tetracene, significant hybridisations occur with the very-close-lying in-phase and antiphase molecular bending vibrations.

To understand the evolution of the energies of the modes with acene length, it is again useful to consider a simple mechanical model: in a spring–mass system, the (angular) frequency, *ω*, depends on the mass, *M*, and the effective spring constant, *k*_eff_, as

2 *ω*^2^ = *k*_eff_*M*^−1^

As the masses of the different acenes are known, eqn (2) can be used to determine the effective force constants for the different TRIMMs. These are plotted in Fig. 2(h) as a function of the associated molecular mass values. Notably, the effective intermolecular force constant for the highest TRIMM (***a***_1_/normal) scales linearly with the length and molecular mass (see the linear fit through the origin in Fig. 2(h)). This is plausible, as for a vibration in which primarily the distance between neighbouring acenes is varied, the forces (determined by van der Waals attraction and Pauli repulsion) should scale with the intermolecular overlap. Thus, longer molecules show stronger interactions and larger intermolecular force constants for this type of motion. According to eqn (2), a simultaneous linear increase in molecular mass and intermolecular force constant results in a constant vibrational frequency, as observed in Fig. 1(e) for the ***a***_1_/normal TRIMM.

The ***a***_2_/short TRIMM corresponds to a vibration that primarily induces a change in molecular overlap along the entire length of the molecule, as shown in Fig. 2(f). This suggests that also for the ***a***_2_/short TRIMM the effective force constant should increase essentially linearly with the acene length and, thus, with the molecular mass, and the associated frequencies should again stay largely constant. This is indeed observed in Fig. 2(f) and (e) for all acenes apart from benzene for which the force constant is higher than a linear fit through the origin would suggest. This then causes the distinctly higher frequency of the respective mode for benzene shown in Fig. 1(e). We attribute this to peculiarities of the benzene structure, especially the significant increase of the angle between the short molecular axes of the two molecules per unit cell, which results in a steeper increase of intermolecular interactions upon alternatingly displacing the rows of molecules along ***a***_2_ (see Section S3, including the provided animations, ESI†).

Finally, for the lowest-energy TRIMM the associated displacement along the long molecular axis should induce at most a weak length dependence of changes in the geometric overlap between neighbouring molecules, as here primarily the overlap of the outermost rings is modified (see Fig. 2(e)). This suggests a length-independent effective force constant, which is indeed observed with the exception of tetracene, for which the force constant is somewhat smaller than expected. Consequently, the vibrational frequency is expected to drop with the one over the square root of the molecular mass, fully consistent with Fig. 1(e), with tetracene again being an outlier here with a too small frequency. The reason for that is not fully understood, but it should be mentioned that tetracene is the first system in the series for which a fundamental change in the tilting direction of the two molecules is observed (see the above description of the geometry and Section S3, ESI†). In addition, tetracene lies at the transition point between systems in which the ***a***_3_/long TRIMM couples with an antiphase (for the shorter acenes) *vs.* an in-phase bending motion (for pentacene).

The dispersions of the TRIMM bands are rather similar for benzene, naphthalene, anthracene and pentacene-I with band widths of around 0.2–1.2 THz (the largest band widths are found for the ***a***_1_/normal TRIMMs). A fully quantitative determination of the band widths is, however, complicated by the occurrence of avoided crossings. The rather significant band dispersion for TRIMMs is not unexpected considering that the relative phase of translational motions in neighbouring unit cells (expressed by the wavevector ***q***) strongly impacts the relevant intermolecular distances and, thus, also the intermolecular interactions and the frequencies of the vibrations.

The last class of low-frequency vibrations is the in-phase and antiphase rotations of the molecules, which in the following will be referred to as **r**otational **r**igid **i**nter**m**olecular **m**odes (RRIMMs). As shown in Fig. 1(e), the two highest RRIMM bands correspond to rotations around an axis largely parallel to the ***a***_3_ direction and, thus, largely around the long molecular axes especially for naphthalene to tetracene (see animations in the ESI†). For benzene, the rotational axis is strongly inclined and in the two pentacene polymorphs there is a quite significant hybridisation with very close-lying intramolecular modes (see Fig. 1(d, e) and Section S9.2, ESI†). For ***a***_3_/long-axis rotational modes, one would expect the moment of inertia (as the quantity analogous to the molecular mass in eqn (2) for a rotational mode) to increase linearly with molecular length and a similar evolution would be expected for the restoring torque. Indeed, when plotting the effective rotational force constant (again in analogy to eqn (2)) as a function of the moment of inertia around the long molecular axis (see Section S7.3, ESI†), one observes a linear dependence similar to the ***a***_3_/long TRIMM. This is consistent with the rather system-independent frequencies of the in-phase and antiphase ***a***_3_/long RRIMMs. Benzene is again an outlier, especially for the in-phase mode, which we primarily attribute to the strong inclination of the rotational axis relative to the long molecular axis mentioned above. The variations in frequency are, however, more pronounced than for the ***a***_3_/long TRIMM. This is insofar not surprising, as molecules tilt differently in the different systems: for 1A, 2A, and 3A the tilting directions of the long molecular axes of the two molecules are different with rather significantly varying tilt angles; conversely, both molecules tilt in the same direction for 4A and 5A (see above and Section S3, ESI†). This then causes some variation of the orientation of the rotational axis between the different systems.

The situation becomes even more involved for rotations, which are nominally around the short molecular axes or around axes normal to the molecular plane (*i.e.*, around axes nominally parallel to ***a***_2_ and ***a***_1_). There, one faces two major problems: first, it is difficult to formulate expectations based on classical models, as the moments of inertia and the restoring forces for such vibrations are expected to have a strongly non-linear dependence on the molecular size. The latter is primarily due to the rather different displacement amplitudes for the outer rings in the different systems, which then cause large differences in the intermolecular interactions. Second, an in-depth inspection of the associated atomic displacements shows that the orientations of the rotational axes for these RRIMMs vary strongly between the systems. Therefore, we provide a detailed discussion of the ***a***_1_/normal and ***a***_2_/short RRIMMs only in Section S7.3 (ESI†).

As far as the observed band dispersions are concerned, the bands associated with the RRIMMs are not necessarily flat, despite the considerably reduced participation ratios. This can again be attributed to the significant impact of the vibrational displacements on the intermolecular interactions triggering a strong dependence of the frequencies on the phase shift (and, thus, on ***q***). Still, in view of the significantly changing structures of the bands for the different acenes it appears hardly possible to extract general trends. Therefore, we will focus the subsequent discussion of group velocities as a measure of band dispersions on the particularly relevant acoustic phonons and only at the end of the next section will return to higher-lying, optical bands.

### Group velocities

Besides their fundamental relevance, group velocities are also of distinct practical importance, as they significantly impact all energy-transport related phonon properties (such as the thermal conductivity). Their full analysis is, however, quite involved, as the group velocity, ***v***_g_, is a three-component vector for each phonon. Thus, in the following we will resort to discussing (vector) norms of the group velocity, *v*_g_, and, as a first step, will focus on acoustic phonons in the long-wavelength limit (*i.e.*, at small wave vectors, ***q***). These are representative of the situation for a large fraction of reciprocal space, as the acoustic bands display a linear dispersion (and, thus, a constant group velocity) over a rather wide frequency range (see Fig. 1(a–c)).

**Fig. 3:**
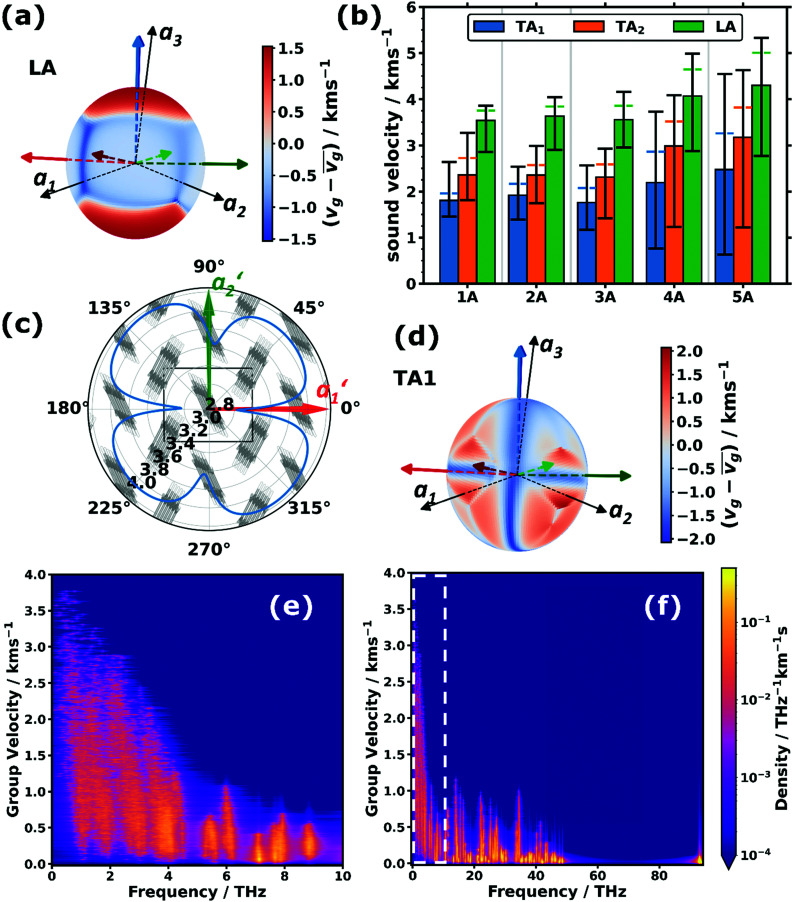
(a and d) Spatial distributions of the variations of the sound velocities, *v*_g_, projected onto unit spheres for the (a) (primarily) longitudinal acoustic (LA) and (d) lower (primarily) transverse acoustic (TA_1_) modes in pentacene relative to their arithmetic mean values, *v̄*_g_ (4.30 km s^−1^ and 2.47 km s^−1^). To facilitate the orientation in space, the directions of the lattice vectors (***a***_1_, ***a***_2_, and ***a***_3_) are indicated by the black arrows. In addition, the long molecular axes (blue arrows), the short molecular axes (red arrows) and the normal vectors to the π-planes (green arrows) of the two molecules per unit cell are indicated. The three arrows with a slightly darker shade belong to one molecule, while the lighter arrows belong to the other molecule. (b) Average sound velocities of the LA and TA_1/2_ phonon branches (bars) for the studied acenes from benzene (1A) to pentacene (5A). The “error margins” indicate the minimum and maximum observed sound velocity values of the respective type of phonon band on the sampled sphere. The distances between the upper edges of the bars and the horizontal coloured dashed lines correspond to the standard deviations of the distributions of sound velocities of the respective type. (c) Polar plot of the LA sound velocity (units: km s^−1^) in pentacene in a plane perpendicular to the lattice vector ***a***_3_ overlaid with the crystal structure seen along this direction. The projections of the remaining two lattice vectors in this plane are labelled with ***a***_1_′ and ***a***_2_′. (e and f) Two-dimensional densities of states per group velocity and frequency for pentacene. The white dashed box in (f) indicates the region of the expanded area shown in (e).

Of particular interest are the longitudinal acoustic (LA) bands, which typically display the largest group velocities in a solid and which also correspond to the (longitudinal) sound velocities. At this point it is worth mentioning that purely longitudinal or transverse phonon polarisations with entire lattice planes moving in phase are observed only in high-symmetry directions of highly symmetric crystals, while, in general, the polarisations have a mixed longitudinal–transverse character.^78,79^ Nevertheless, in the following, we will refer to longitudinal acoustic and transverse acoustic (TA) modes, whenever they have a primarily longitudinal or transverse character. Prior to discussing the absolute magnitudes of the LA group velocities and their evolution with molecular length, it is interesting to analyse their directional dependence. Fig. 3(a) shows the directional dependence of the deviation of *v*_g,LA_ from its mean value of 4.30 km s^−1^ for 5A (equivalent plots for the other acenes are shown in Section S8, ESI†). This schematic testifies to a pronounced anisotropy of *v*_g,LA_, which is not surprising, considering the highly anisotropic crystal structures of the acenes. As quantified in Fig. 3(b), *v*_g,LA_ in 5A varies between 2.77 and 5.33 km s^−1^, *i.e.*, by nearly a factor of 2. It is largest in a direction essentially parallel to the long molecular axis (and the ***a***_3_ axis). This can again be explained by a classical model. The group velocity of acoustic phonons in high-symmetry directions in the long wavelength limit is approximately given by^78–80^3
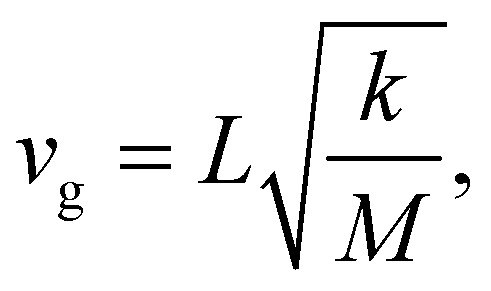
with *k* representing the force constant describing the interactions between the oscillating objects, and *M* and *L* representing their mass and distance, respectively. The latter is particularly large along the ***a***_3_ axis with the effect being most pronounced for 5A. The distance dependence can be rationalised by the fact that *L* represents the period of the weak intermolecular van der Waals bonds that are compressed and expanded during wave propagation and, thus, delay the speed of elastic energy migration. Moreover, an in-depth analysis of the displacements shows that at higher ***q***-values (*i.e.*, for large phase shifts between neighbouring unit cells) the phonons also comprise some compression and expansion of the particularly stiff covalent C–C bonds within the molecules. Group velocities in directions parallel to the short molecular axis and perpendicular to the π-planes are intermediate (see the red and green arrows in Fig. 3(a)), while particularly low group velocities are found in directions parallel to ***a***_1_ and ***a***_2_. This also applies to directions parallel to their projections into the plane perpendicular to ***a***_3_ (termed ***a***_1_′ and ***a***_2_′), which is illustrated in Fig. 3(c). The intermolecular distances between the molecules and their periodic replica in these directions (as a measure of *L*) are comparably small and, considering the arrangement of the molecules in the plane perpendicular to ***a***_3_ (see Fig. 3(c)), it is, indeed, plausible that a longitudinal compression of the unit cell along ***a***_1_′ and ***a***_2_′ would be energetically not very costly (*i.e.*, the associated force constant would be comparatively small). This is at least suggested by the animations of the displacements for wave propagation in the ***a***_1_′- and ***a***_2_′-directions (see the animation in the ESI†). In the ***a***_2_-direction, they primarily involve π-planes gliding past each other, and in the ***a***_1_-direction they are determined by motions of comparably distant and only partially overlapping π-planes towards and away from each other.

The above-described anisotropies prevail for the shorter acenes, albeit with a decreasing magnitude of the directional variation of *v*_g,LA_ (see Fig. 3(b) and the polar plots in Section S8, ESI†). The only exception is benzene, for which the maximum of *v*_g,LA_ is no longer parallel to the molecular axis, which is defined as the “long axis” in accordance with the longer acenes. This is a consequence of the molecule not having its largest extent in that direction (see the discussion in Section S2, ESI†).

Based on the above arguments, one can also understand the variation of *v*_g_ with molecular length (see the “error margins” of the green bars in Fig. 3(b)). The minimum group velocities (the lower margin) remain essentially constant, as the situation does not change very significantly as a function of the molecular length for wave propagation close to the ***a***_1_***,a***_2_-plane. Here, the force constants and the molecular masses both increase linearly with molecular length, analogous to the above explanation for the system-independent *Γ*-point energy of the ***a***_1_/normal and ***a***_2_/short TRIMMs. These modes involve conceptually similar displacements as the LA modes in the ***a***_1_- and ***a***_2_-directions (albeit of the two molecules within the unit cell relative to each other rather than of molecules in neighbouring cells). Conversely, the increase of the maximum (and concomitantly average) values of *v*_g_ can be explained by an increasing intermolecular distance in ***a***_**3**_ direction, which enters linearly into eqn (3), and which is only partly compensated by the increasing molecular mass entering only to the power of −1/2.

As expected, the group velocities associated with the transverse acoustic (TA) modes are significantly smaller than for the longitudinal modes. Interestingly, one typically observes similar minimum, maximum and average values for 1A, 2A, and 3A, while for longer acenes, the minimum values of *v*_g,TA1_ and *v*_g,TA2_ drop and the maximum values increase, such that the anisotropy becomes particularly large. For example, for the TA_1_ mode in 5A the ratio between the minimum and maximum values of *v*_g,TA1_ becomes as large as ∼7.1. Moreover, the directions of minimum and maximum group velocities appear in fundamentally different directions than for the longitudinal modes. This is exemplarily shown for the TA_1_ mode of 5A in Fig. 3(d), for which the group velocities are particularly small for wave propagation along the short and long molecular axes as well as perpendicular to the π-planes (the corresponding plots for the TA_2_ mode and for the other acenes can be found in Section S8, ESI†).

The above considerations exclusively concern the acoustic phonons in the long wavelength limit. However, already the bands shown in Fig. 1(a–c) indicate that in acenes there are also several significantly dispersing optical phonon bands. This applies particularly to the low-frequency region, as shown in Fig. 3(e) for 5A. Interestingly, Fig. 3(f) shows that there are also rather significantly dispersing bands at even higher frequencies (up to ∼35 THz). Examples for high-lying optical bands with maximum *v*_g_ values above 1 km s^−1^ in pentacene comprise intramolecular bending modes around the molecules’ long axes (at ∼13.5 THz and ∼13.7 THz at *Γ*), out-of-plane bending of the hydrogen atoms in an alternating up-down fashion (at ∼21.7 THz at *Γ*), and in-plane bending of the C–H bonds (at ∼34.5 THz at *Γ*) with the six H atoms in the centre of the molecule bending in one direction, while the hydrogens at both ends bend in the other. For all these modes one can understand that intermolecular interactions play a non-negligible role, such that the phase shifts of the displacements in neighbouring unit cells have an impact on the interaction energy such that the phonon frequency becomes strongly ***q***-dependent, which then causes a comparatively large group velocity. Similar considerations apply to the other acenes as shown in Section S4.4 (ESI†), which also contains a brief discussion of the densities of states per group velocity.

### Phonon contribution to the heat capacity

**Fig. 4:**
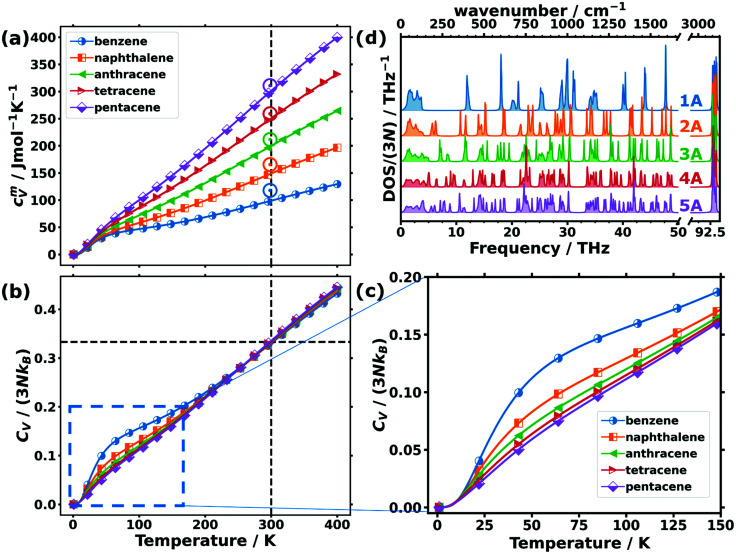
(a) Molar heat capacity and (b) heat capacity per unit cell normalised by the classical Dulong–Petit limit (3*Nk*_B_) as a function of temperature for the studied organic semiconductor crystals. The vertical dashed lines indicate the temperature of 300 K, while the horizontal line emphasises the common value in the normalised heat capacities of ∼0.33 observed at that temperature. Note that the upper limit of the shown temperature range already exceeds the melting point of benzene (at ∼279 K) and naphthalene (∼353 K) at ambient pressure.^81^ The open circles in (a) indicate the experimentally determined values for the molar constant-pressure heat capacity for benzene,^82,83^ naphthalene,^83,84^ anthracene,^83,85^ tetracene,^86^ and pentacene^86^ in the corresponding colours. (c) Expanded region of panel (b) indicated by the dashed blue rectangle. (d) Density of states (DOS) normalised with the number of bands (3*N*) of the studied acenes: benzene (1A) to pentacene (5A).

While the above discussion focused on low-frequency phonons, there are also quantities for which higher-frequency phonons play a role. This is particularly true for thermodynamic properties at room temperature or at elevated temperatures, at which higher-lying phonon states also become occupied. An important example of such a thermodynamic property is the phonon contribution to the molar heat capacity at constant volume, *c*_V_^*m*^. This quantity (calculated from the full phonon band structures) is shown in Fig. 4(a) as a function of temperature for the studied OSCs together with the experimental values for the molar heat capacities at constant pressure, *c*_p_^*m*^, at room temperature. The simulations fully confirm the experimental trend with the absolute values being somewhat smaller in the calculations than in the measurements. This is not unexpected, as *c*_p_^*m*^ is generally larger than *c*_V_^*m*^, since in *c*_V_^*m*^ the effect of thermal expansion is not considered.^80^

For comparing the different acenes on an “equal footing” it is useful to convert the molar heat capacity, *c*_V_^*m*^, to the heat capacity per unit cell, *C*_V_ (by dividing *c*_V_^*m*^ by Avogadro's number and multiplying it by the number of molecules per unit cell). In the classical Dulong–Petit limit (*i.e.*, for the temperature *T* → ∞) *C*_V_ amounts to4
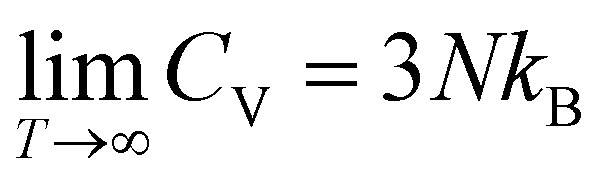
Here, *N* is the number of atoms contained in the unit cell, and *k*_B_ is the Boltzmann constant. Notably, the normalised heat capacity per unit cell, *C*_V_/(3*Nk*_B_), ranges between 0 and 1 regardless of the number of atoms or molecules per unit cell. It is plotted in Fig. 4(b) as a function of temperature for the different acenes with an expanded version of the low-temperature region in Fig. 4(c).

Starting from *C*_V_/(3*Nk*_B_) = 0 at 0 K (in agreement with the third law of thermodynamics), the heat capacity rises continuously with *T*, concomitant with an increasing occupation of the higher-frequency states.^78,80^ At room temperature, the heat capacity is still far from its classical limit (*C*_V_/(3*Nk*_B_) = 1), which is a consequence of the many high-frequency modes occurring in acenes (with the C–H stretching vibrations at ∼93 THz as the most extreme example). Interestingly, at room temperature, *C*_V_/(3*Nk*_B_) is virtually identical for all the systems and amounts to ∼0.33. This implies that the nature of the atoms contained in the molecules and especially the carbon-to-hydrogen ratio (which is 1.0 for benzene and 1.6 for pentacene) play essentially no role for that quantity. Consequently, all that really counts for the molar heat capacity is the total number of atoms per molecule (see Section S10 for more details, ESI†), such that the increase of the molar heat capacity with the length of the acenes at room temperature (see Fig. 4**(a)**) is merely a consequence of the larger numbers of atoms in the bigger molecules. The explanation for this lies in the (normalised) phonon densities of states shown in Fig. 4(d). They show that with increasing complexity of the molecules, the additional intramolecular vibrations are relatively uniformly distributed over the entire frequency range. Variations in the normalised heat capacity would occur only if the density of states in a specific frequency region differed notably for a given material. This is, in fact, the case in the low-frequency region, where there are twelve intermolecular modes per unit cell, independent of the length of the molecules (see above). Consequently, for the shorter acenes the weight of the intermolecular modes in that region increases relative to the entire DOS. For the longer acenes, this is only partially compensated by the decrease of the frequencies of the first intramolecular modes with molecular length as discussed above. As a result, at low temperatures (where mostly the low-frequency phonons count) the normalized heat capacity per unit cell is significantly increased, especially in benzene and also in naphthalene (see Fig. 4(c)).

## Conclusions

This article uses state-of-the art density-functional-theory-based simulations to explain the relationship between the phonon band structures of acenes and the respective structures of the molecules and crystals. The discussion focuses on the low-frequency region (up to ∼7 THz), which is particularly relevant for heat and charge-transport processes. It is dominated by intermolecular vibrations (*i.e.*, motions of the two molecules per unit cell as more or less rigid objects) and by intramolecular modes characterised by distortions of the entire molecular backbones. At first glance, the calculated phonon band structures appear to be highly complex with no indications for apparent structure–property relationships. An in-depth analysis of the individual bands, however, allows us to distinguish between phonons associated with intramolecular in-phase and out-of-phase bending and torsion modes, as well as intermolecular “translational and rotational rigid intermolecular modes” (TRIMMs and RRIMMs). The evolution of the intramolecular modes and of the TRIMMS with the acene length can be rationalized very well using simple classical models of bending beams and (torsional) spring–mass oscillators, and the details of the arrangements of the molecules in the different acene crystals typically play only a minor role. The simple models allow us to rationalise, why the frequencies of the considered intramolecular modes drop with acene length and why such a behaviour is observed only for certain TRIMMs. The situation becomes less clear for the RRIMMS, especially for those associated with rotations around axes other than the long molecular axis. This is primarily attributed to the system-dependent orientations of the respective rotational axes due to variations in the arrangements of the acene molecules in the unit cells.

As far as the group velocities of the acoustic phonons are concerned, we observe pronounced anisotropies with the highest group velocities reaching values of up to 5.33 km s^−1^. For the longitudinal modes these anisotropies can again be rationalised based on classical models and the crystal structure. This also applies to the evolution of the maximum and minimum longitudinal group velocities. Unfortunately, the situation becomes much more complex for the (primarily) transverse modes. Considering the band dispersions and group velocities of the optical modes above 2 THz, one finds values of up to ∼2.8 km s^−1^, which is only a factor of ∼1.9 smaller than the fastest acoustic phonons. Notably, even for frequencies up to 35 THz, bands with group velocities beyond 1 km s^−1^ are observed, which can be rationalised by the nature of the involved vibrations. Finally, as an example of a thermodynamic quantity, we show how the phonon properties correlate with the phonon heat capacity as a function of temperature and chain length.

In conclusion, the presented results show how analogies to classical macroscopic oscillators can be exploited to estimate and explain the shifts in phonon frequencies and the associated change in physical properties in a series of related molecular crystals. These considerations are, by no means, restricted to the here-studied series of acenes but can also be extrapolated to other organic semiconductors. Thus, we expect the above observations and their explanations to serve as a foundation on which the future systematic investigation of phonon properties of more complex organic semiconductor crystals can be built.

## Author contributions

T. K. and E. Z. conceptualised the present study of the structure–property relationships in phonon-derived properties of organic semiconductor crystals. T. K. conducted all the quantum–mechanical simulations as well as the formal analysis of the results, including coding of the necessary analysis software tools. T. K. wrote the original draft of the manuscript and prepared all the figures, which were revised by T. K. and E. Z., who was also responsible for the supervision of the project.

## Conflicts of interest

There are no conflicts to declare.

## Supplementary Material

TC-010-D1TC04708F-s001

TC-010-D1TC04708F-s002

TC-010-D1TC04708F-s003

TC-010-D1TC04708F-s004

TC-010-D1TC04708F-s005

TC-010-D1TC04708F-s006

TC-010-D1TC04708F-s007

TC-010-D1TC04708F-s008

TC-010-D1TC04708F-s009

TC-010-D1TC04708F-s010

TC-010-D1TC04708F-s011

TC-010-D1TC04708F-s012

TC-010-D1TC04708F-s013

TC-010-D1TC04708F-s014

TC-010-D1TC04708F-s015

TC-010-D1TC04708F-s016

TC-010-D1TC04708F-s017

TC-010-D1TC04708F-s018

TC-010-D1TC04708F-s019

TC-010-D1TC04708F-s020

TC-010-D1TC04708F-s021

TC-010-D1TC04708F-s022

TC-010-D1TC04708F-s023

TC-010-D1TC04708F-s024

TC-010-D1TC04708F-s025

TC-010-D1TC04708F-s026

TC-010-D1TC04708F-s027

TC-010-D1TC04708F-s028

TC-010-D1TC04708F-s029

TC-010-D1TC04708F-s030

TC-010-D1TC04708F-s031

TC-010-D1TC04708F-s032

TC-010-D1TC04708F-s033

TC-010-D1TC04708F-s034

TC-010-D1TC04708F-s035

TC-010-D1TC04708F-s036

TC-010-D1TC04708F-s037

TC-010-D1TC04708F-s038

TC-010-D1TC04708F-s039

TC-010-D1TC04708F-s040

TC-010-D1TC04708F-s041

TC-010-D1TC04708F-s042

TC-010-D1TC04708F-s043

TC-010-D1TC04708F-s044

TC-010-D1TC04708F-s045

TC-010-D1TC04708F-s046

TC-010-D1TC04708F-s047

TC-010-D1TC04708F-s048

TC-010-D1TC04708F-s049

TC-010-D1TC04708F-s050

TC-010-D1TC04708F-s051

TC-010-D1TC04708F-s052

TC-010-D1TC04708F-s053

TC-010-D1TC04708F-s054

TC-010-D1TC04708F-s055

TC-010-D1TC04708F-s056

TC-010-D1TC04708F-s057

TC-010-D1TC04708F-s058

TC-010-D1TC04708F-s059

TC-010-D1TC04708F-s060

TC-010-D1TC04708F-s061

TC-010-D1TC04708F-s062

TC-010-D1TC04708F-s063

TC-010-D1TC04708F-s064

TC-010-D1TC04708F-s065

TC-010-D1TC04708F-s066

TC-010-D1TC04708F-s067

TC-010-D1TC04708F-s068

TC-010-D1TC04708F-s069

TC-010-D1TC04708F-s070

TC-010-D1TC04708F-s071

TC-010-D1TC04708F-s072

TC-010-D1TC04708F-s073

TC-010-D1TC04708F-s074

TC-010-D1TC04708F-s075

TC-010-D1TC04708F-s076

TC-010-D1TC04708F-s077

TC-010-D1TC04708F-s078

TC-010-D1TC04708F-s079

TC-010-D1TC04708F-s080

TC-010-D1TC04708F-s081

TC-010-D1TC04708F-s082

TC-010-D1TC04708F-s083

TC-010-D1TC04708F-s084

TC-010-D1TC04708F-s085

TC-010-D1TC04708F-s086

TC-010-D1TC04708F-s087

TC-010-D1TC04708F-s088

TC-010-D1TC04708F-s089

TC-010-D1TC04708F-s090

TC-010-D1TC04708F-s091

TC-010-D1TC04708F-s092

TC-010-D1TC04708F-s093

TC-010-D1TC04708F-s094

TC-010-D1TC04708F-s095

TC-010-D1TC04708F-s096

TC-010-D1TC04708F-s097

TC-010-D1TC04708F-s098

TC-010-D1TC04708F-s099

TC-010-D1TC04708F-s100

TC-010-D1TC04708F-s101

TC-010-D1TC04708F-s102

TC-010-D1TC04708F-s103

TC-010-D1TC04708F-s104

TC-010-D1TC04708F-s105

TC-010-D1TC04708F-s106

TC-010-D1TC04708F-s107

TC-010-D1TC04708F-s108

TC-010-D1TC04708F-s109

TC-010-D1TC04708F-s110

TC-010-D1TC04708F-s111

TC-010-D1TC04708F-s112

TC-010-D1TC04708F-s113

TC-010-D1TC04708F-s114

TC-010-D1TC04708F-s115
