# Supplementary material for: Discovering structure–property relationships for the phonon band structures of hydrocarbon-based organic semiconductor crystals: the instructive case of acenes
Source: J Mater Chem C Mater. 2021 Oct 15;10(7):2532–43. doi: 10.1039/d1tc04708f (PMC8852262; doi:10.1039/d1tc04708f)
Supplement: TC-010-D1TC04708F-s115 [file TC-010-D1TC04708F-s115.pdf]

Electronic Supplementary Material (ESI) for Journal of Materials Chemistry C.
This journal is © The Royal Society of Chemistry 2021

Electronic Supplementary Information

Discovering Structure-to-Property Relations in Phonon Band Structures of Hydrocarbon-Based Organic Semiconductor Crystals: The Instructive Case of Acenes

*Tomas Kamencek and Egbert Zojer**

Table of Contents

S1. Data availability 2
S2. Experimental starting geometries 2
S3. Details on the structural similarities and differences among the discussed systems 2
S4. Methodological details 9
S4.1 Further settings for the density functional theory calculations 9
S4.2 Geometry and unit cell optimisations 10
S4.3 Phonon calculations 10
S4.4 Densities of states per frequency and per group velocity 11
S4.5 Participation ratios 16
S5. Animations of phonon modes 17
S5.1 Γ-phonons 17
S5.2 Acoustic phonons close to Γ 21
S6. Phonon band structures and first Brillouin zones 21
S7. Details on the classical models to describe the evolution of Γ-frequencies 25
S7.1 Intramolecular bending model 25
S7.2 Intramolecular torsion model 26
S7.3 Rotational rigid intramolecular modes model 28
S8. Sound velocities (long-wavelength limit) 32
S9. Effects of Polymorphism 36
S9.1 Orthorhombic benzene 36
S9.2 Pentacene polymorph II 38

S10. Molar heat capacities 41
S11. Comparison of low-frequency intramolecular between the crystals and the isolated molecules 43

S1. Data availability

The simulation input and output required to reproduce the presented results are publicly available in the NOMAD database.[1]

S2. Experimental starting geometries

The crystal structures used as starting points for the geometry optimisations are listed in Table S 1. Additionally, the table shows the identifiers and deposition numbers of the crystal structures of the Cambridge Structural Database (CSD).[2]

Table S 1: Experimental crystal structures used as starting points for the DFT geometry optimizations.

Molecule/Phase	Abbreviation	CSD identifier and deposition number of the starting geometry	Reference
Benzene/ Orthorhombic Polymorph I	1A-o	BENZEN01 / 1108750	3
Benzene/ Monoclinic Polymorph III	1A	BENZEN03 / 1108752	4
Naphthalene	2A	NAPHTA31 / 600182	5
Anthracene	3A	ANTCEN14 / 1103074	6
Tetracene	4A	TETCEN01 / 114446	7
Pentacene/Polymorph I	5A	PENCEN / 1230799	8
Pentacene/Polymorph II	5A-II	PENCEN04 / 170187	9

S3. Details on the structural similarities and differences among the discussed systems

Table S 2 contains the main parameters of the unit cells of the studied systems. One observes rather significant variations of the area of the basal plane, the aspect ratios, and also the nearest-neighbour distances as well as the distances to the nearest symmetry-equivalent molecules (which are given by $\boldsymbol{a_1}$ and $\boldsymbol{a_2}$, respectively). Also, some other variations stand out, like the particularly

small (large) area of the basal plane for monoclinic benzene (tetracene) and the generally smaller aspect ratios for the triclinic systems (which also result in smaller nearest-neighbour distances), but by and large, the variations in the geometric parameters appear not to be particularly systematic. Thus, in the following table, several geometric parameters are collected that characterise the arrangement of neighbouring molecules in the unit cell. Note that in order to be consistent with the unit cells of the longer acenes (see caption of Table S 2), the long/short molecular axis as well as the lattice vectors must be reassigned for 1A, 4A, and 5A-II. This reassignment is explained for the most complex case of 1A by comparing the crystal structure of 1A and 2A in Figure S 3.

Table S 2: Unit cell-related parameters of the studied systems: The table contains the lengths of the lattice vectors and the angle between them. The $\boldsymbol{a_1},\boldsymbol{a_2}$ *plane corresponds to the plane in which the acene molecules are arranged;* $\boldsymbol{a_1}$ *is always chosen such that that it is the longer of the two vectors;* $\boldsymbol{a_3}$ *is the direction that is (more or less) parallel to the long molecular axis. For the sake of consistency, this naming convention is applied to all systems, even in cases in which the order of the unit cell vectors is changed in the respective files in the Cambridge Structural Database (CSD); i.e. for 4A and 5A-II the* $\boldsymbol{a_2}$ *axes of the CSD become a1 and the -* $\boldsymbol{a_1}$ *axis becomes* $\boldsymbol{a_2}$*. The situation is even more involved for monoclinic benzene, where the* $\boldsymbol{a_2}$ *axes of the CSD becomes –* $\boldsymbol{a_2}$ *and the* $\boldsymbol{a_1}$ *and* $\boldsymbol{a_3}$ *axes become –* $\boldsymbol{a_3}$ *and -*$\boldsymbol{a_1}$. The reason for that is illustrated *in Figure S 3. Other than that, the unit-cell definitions from the CSD are kept, even if that means that the definitions of the* $\boldsymbol{a_1}$ *vectors in A4 and A5-II are not consistent with the other systems, as manifested by smaller than 90°* α_3 *values and as exemplarily shown in Figure S 1.* α_1 *is the angle between* $\boldsymbol{a_3}$ *and* $\boldsymbol{a_2}$*,* α_2 *the angle between* $\boldsymbol{a_1}$ *and* $\boldsymbol{a_3}$*, and* α_3 *the angle between* $\boldsymbol{a_2}$ *and* $\boldsymbol{a_1}$*.* $A_{\boldsymbol{a1a2}}$ *is the area of the basal plane of the unit cell, and AR the aspect ratio of the two vectors* $\boldsymbol{a_1}$ *and* $\boldsymbol{a_2}$*.* d_{nn}*, the nearest neighbour distance is defined as half of the length of the shorter diagonal of* $A_{\boldsymbol{a1a2}}$*.*

	**crystal class**	$\boldsymbol{a_1}$ / Å	$\boldsymbol{a_2}$ / Å	$\boldsymbol{a_3}$ / Å	α_1 / °	α_2 / °	α_3 / °	$A_{\boldsymbol{a1a2}}$ / Å^2	***AR***	d_{nn} / Å
1A-o*)	orthorhombic	6.756	7.318	9.288	90.0	90.0	90.0	49.44	1.08	4.98
1A	monoclinic	7.768	5.558	5.632	90.0	111.2	90.0	**43.18**	1.40	4.776
2A	monoclinic	8.078	5.903	8.622	90.0	124.2	90.0	47.69	1.37	5.003
3A	monoclinic	8.426	5.930	11.110	90.0	125.4	90.0	49.97	1.42	5.151
4A	triclinic	7.776	6.016	12.911	72.6	77.1	94.5	46.63	**1.29**	4.725
5A	triclinic	7.733	6.071	15.839	103.3	113.1	85.1	46.78	**1.27**	4.708
5A-II	triclinic	7.638	6.271	14.314	89.0	76.8	95.9	47.65	**1.22**	4.685

*)This unit cell contains four molecules.

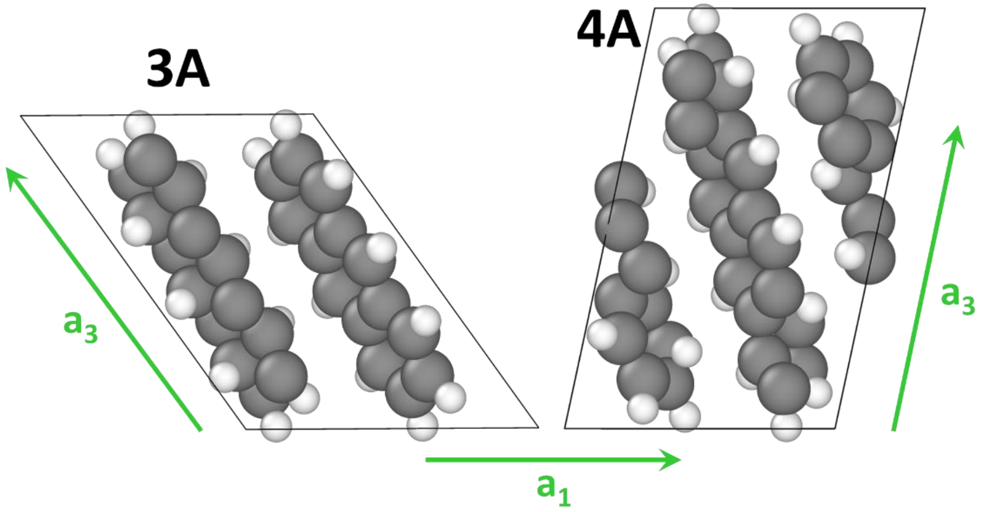

*Figure S 1: Side views of the unit cells of 3A and 4A to illustrate the conceptually different choices of the **a₃** vector that has been made for the data provided in the CSD. Atomic colour coding: C…grey, H…white*

A comparison of the arrangement of the molecules within the unit cell reveals the actual differences between the various acene crystal structures. The most relevant geometrical parameters are summarized in Table S 3 and sketches to better explain their meaning are contained in Figure S 4. Figure S 5 provides top views of the unit cells (with a viewing direction perpendicular to the basal $\boldsymbol{a_1},\boldsymbol{a_2}$-plane). These data allow the following conclusions:

(i) regarding the tilts of the molecules relative to the normal to the $\boldsymbol{a_1},\boldsymbol{a_2}$ plane, there are two groups of acenes: in 1A-3A, the two molecules per unit cell are tilted in different directions, which are symmetrically arranged left above and below the (negative) $\boldsymbol{a_1}$ axis (see 6th column in Table S 3 and Figure S 5). As a result, the angle between the long axes of the molecules is rather large but continuously decreases from 1A (39.4°) to 3A (13.8°). Conversely, for 4A, 5A, and 5A-II, both molecules tilt essentially in the same direction, which is nearly diagonal with respect to the $\boldsymbol{a_1}$ and $\boldsymbol{a_2}$ axes. Consequently, the two molecules are no longer symmetry- equivalent (see Figure S 5) consistent with the triclinic structure of the unit cells.

(ii) This different mode of tilting also results in smaller tilt angles in 4A, 5A, and 5A-II compared to 2A and 3A.

(iii) Monoclinic benzene displays a few additional deviations from the other (short acenes): its tilt angle, θ, is much smaller than that of 2A and 3A, and, more importantly, here the two molecules tilt nearly perpendicular to the $\boldsymbol{a_1}$ axis (i.e., parallel to the $\boldsymbol{a_2}$ axis)

(iv) The angle between the short molecular axes in 1A is much larger than in the longer acenes. Consequently, in 1A the planes of the two molecules per unit cell come to lie close to perpendicular, while they are much less inclined relative to each other in the other acenes.

(v) Finally, two systems also stand out regarding the displacements of equivalent molecules in consecutive layers (see Figure S 4(b)): in benzene, the normal distance between the planes of the molecules amounts to 2.85 Å; i.e., there is a particularly large perpendicular offset, Δ_{perp}, between molecules in consecutive layers, when viewing the structure along the long molecular axis. Conversely, equivalent pentacene molecules in consecutive layers are close to coplanar in 5A-II.

(vi) The values of Δ_{perp} and Δ_{short} also motivate, why the structure of 5A is more consistent with the shorter acenes than that of 5A-II.

Overall, these data show that there are two qualitatively different groups of systems (1A to 3A vs. 4A, 5A and 5A-II), which within the groups share common geometrical features. Moreover, concerning most quantitative parameters, the structure of benzene quite notably differs from that of the longer acenes in its group.

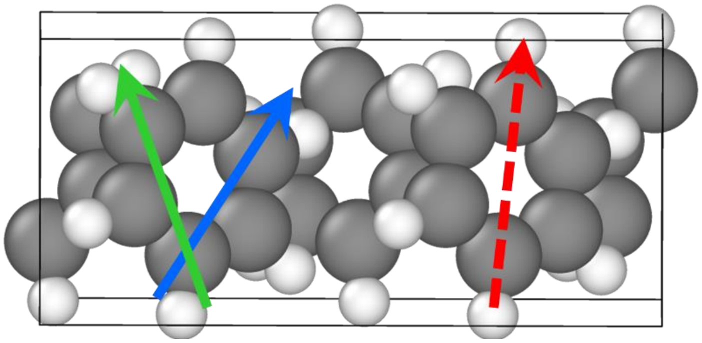

Figure S 2: Ambiguity for choosing the long molecular axis for orthorhombic benzene. The green and blue arrows indicate choices of the long molecular axis that would be consistent with the situations for naphthalene and anthracene, while the dashed red arrow would not be (although it would appear as the natural choice for benzene per se). Atomic colour coding: C…grey, H…white.

*Table S 3: Geometrical parameters describing the arrangement of the molecules within the unit cell. θ_1 and θ_2 denote the angles of the long molecular axes relative to the normal to the basal **a_1,a_2**-plane and ϕ_1 and ϕ_2 are the angles between the projections of the long molecular axes into the basal plane and the **a_1** axis (see sketch in Figure S 4(a)). The orientation of ϕ_1 and ϕ_2 relative to the (negative) **a_1** axis is also summarized in the 6th column of the table. For benzene, the definition of the long molecular axis is a priori not unique, as illustrated in Figure S 2, but a detailed comparison between the structures of benzene and naphthalene reveals that the green arrow in Figure S 2 denotes the most consistent definition of the long molecular axis (see Figure S 3) . φ_{long} and φ_{short} denote the angles between the long and short molecular axes of the two molecules per unit cell. Δ_{perp} and Δ_{short} are the components of the vectors connecting equivalent molecules in consecutive layers (i) perpendicular to the molecular planes and (ii) within the planes parallel to the short molecular axes, as sketched in Figure S 4(b). In cases, in which different shifts for the two molecules in the unit cell are observed, these are separated by a "/". Δ_{perp} also corresponds to the normal distance between the planes defined by equivalent molecules in consecutive layers.*

	θ_1 / °	θ_2 / °	ϕ_1 / °	ϕ_2 / °	**relative to (-)a_1 / °**	φ_{long} / °	φ_{short} / °	Δ_{perp} / Å	Δ_{short} / Å
1A	**19.8**	**19.8**	86.2	273.8	= **±86.2**	**39.4**	**77.9**	**2.58**	**0.40**
2A	30.7	30.7	156.2	203.8	= **±23.8**	23.8	56.4	1.54	1.22
3A	31.5	31.6	166.7	193.3	= **±13.3**	13.8	53.3	1.38	0.82
4A*)	**23.8**	**21.5**	137.9	135.1	**=-46.5/-44.9**	2.6	51.5	1.82/1.23	0.42/1.25
5A*)	**24.4**	**22.7**	222.3	220.2	**=+42.3/+40.2**	1.9	51.1	1.82/1.20	0.43/1.31
5A-II*)	**25.7**	**26.4**	133.5	134.3	**=-46.5/-45.7**	0.8	52.3	**0.81/0.55**	1.14/1.16

* The sign of the angles relative to **-a_1** varies here because of the conceptionally different choices of the **a_3** axes in the CSD for 4A and 5A-II compared to the other crystals, as indicated in Figure S 1; however, this does not imply a conceptual change of the structure. Here, positive angles refer to anticlockwise rotations.

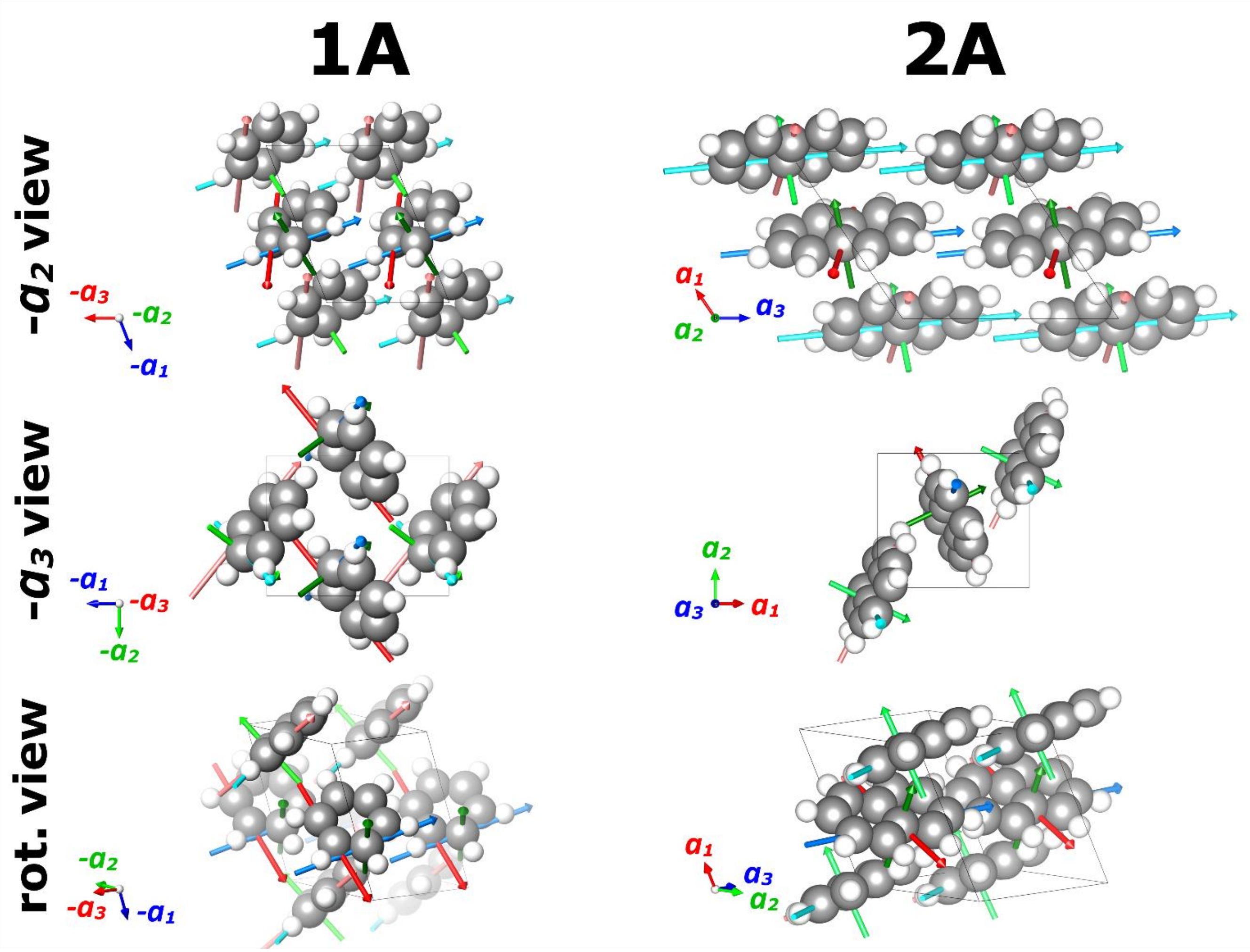

*Figure S 3: Crystal structure of benzene (1A) and naphthalene (2A) shown in different directions. The arrows on the molecules show the long (blue) and short (red) molecular axes as well as directions orthogonal to the π-planes (green). The three arrows with darker shade belong to one of the two molecules per unit cell, the arrows with lighter shade belong to the other molecule. Based on these directions in the longer acenes (e.g., 2A), equivalent directions can be found also in benzene, although the "long" and "short" axes (blue and red arrows) do not correspond to the long and short axis of benzene per se (which for a single ring would pass through the top and bottom atom). Due to the equivalence and similarity of the molecular arrangement, the lattice vectors of the original structure contained in the CSD is changed (**a_1**→ -**a_3**, **a_2** → -**a_2**, **a_3**→-**a_1**) to be consistent with the longer acenes (see above). Atomic colour coding: C…grey, H…white. Visualised using the VESTA 3 code.*[10]

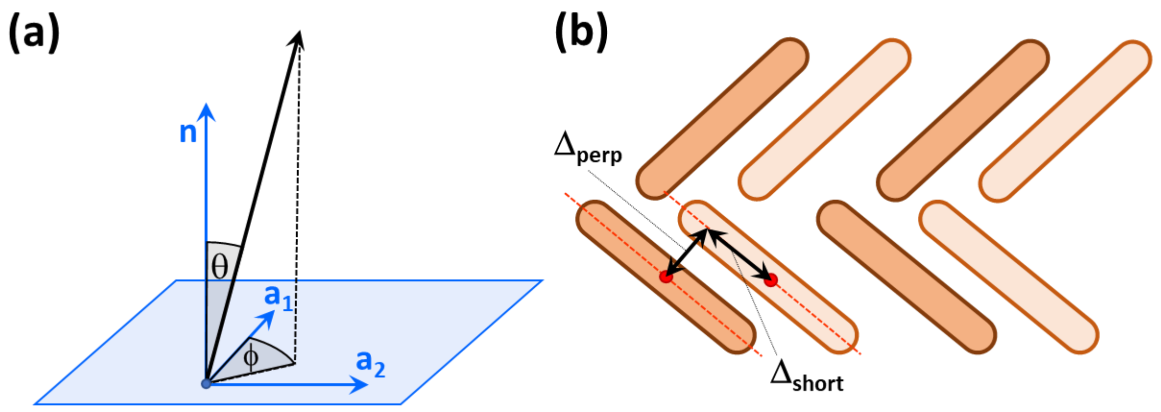

Figure S 4: (a) (a) Schematics defining the angles θ and ϕ for each molecule in the unit cell, where the black arrow corresponds to the long molecular axis of the molecule. (b) Offsets between equivalent molecules viewed along the long molecular axes. Light and dark shaded molecules are in consecutive acene layers and the dashed red lines denote the projections of the respective molecular planes

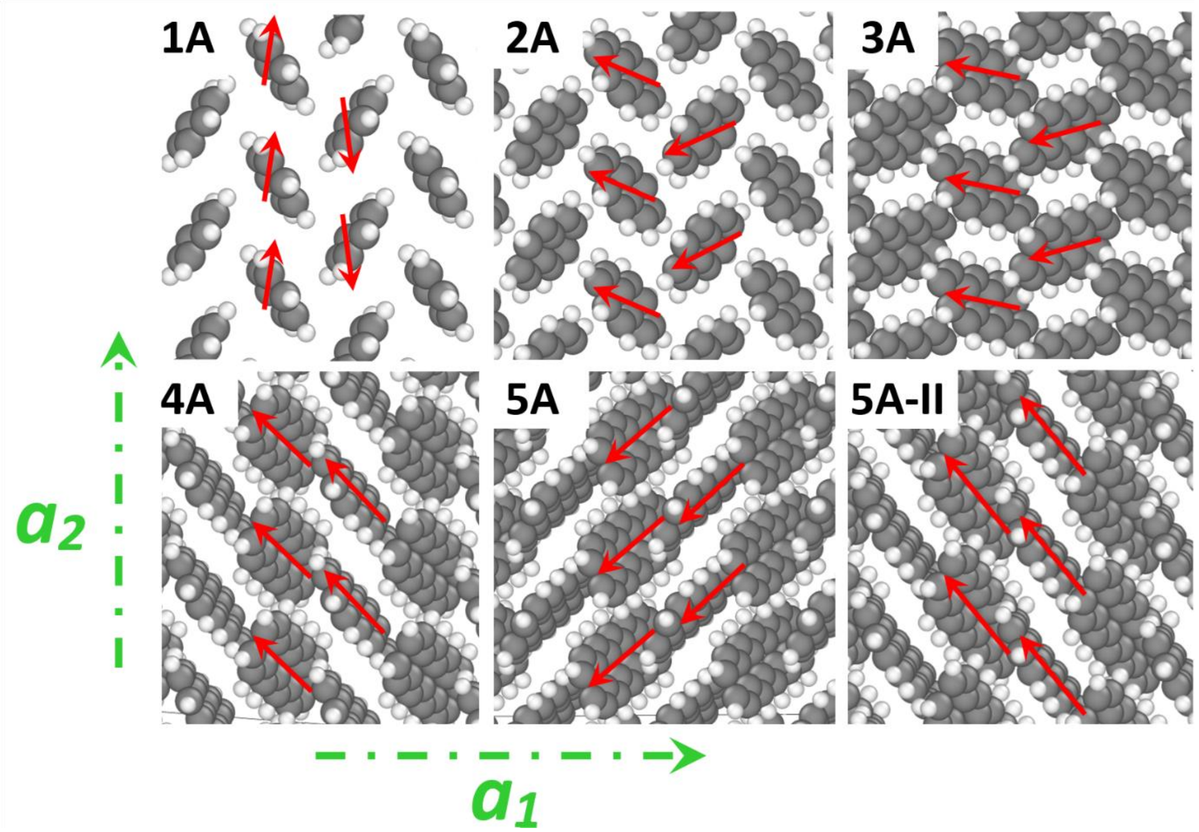

Figure S 5: Structures of the studied series of acenes (1A – 5A-II) viewed in a direction perpendicular to the $\boldsymbol{a_1}$,$\boldsymbol{a_2}$*-plane. The green, dash-dotted arrows are only a guide to the eye to be able to (approximately) assess the orientation of the molecules (i.e., their lengths are not to scale and for 4A, 5A and 5A-II,* $\boldsymbol{a_1}$ *and* $\boldsymbol{a_2}$ *are not orthogonal. The red arrows denote the projections of the long molecular axes to illustrate in which direction the molecules are tilted (see definition of ϕ in Figure S 4(a)). Atomic colour coding: C…grey, H…white.*

S4. Methodological details

S4.1 Further settings for the density functional theory calculations

Besides the general simulation settings described briefly in the main text, the following additional settings for DFT calculations employing the *VASP* code (version 5.4.1) were chosen. The globally fixed parameters include (*VASP* setting tags in parentheses; for details refer to the *VASP* manual[11]): the employed D3-BJ[12,13] *a posteriori* van der Waals correction (`IVDW = 12`), a total energy convergence criterion for the self-consistent field calculation of 10^{-8} eV (`EDIFF = 1E-8`), Gaussian smearing of the electronic states with a width of 0.05 eV (`ISMEAR = 0; SIGMA = 0.05`), and high global precision (`PREC = Accurate`).

The discrete ***k***-meshes of electronic wave vectors were adjusted for each system individually to achieve homogenous sampling of the first Brillouin zones, with the density and the ***k***-mesh being chosen such that the total energies were converged below ~0.5 meV/atom. In these convergence tests, an energy cutoff of 900 eV in all systems in combinations with the ***k***-meshes listed in Table S 4 turned out to achieve the desired level of convergence. These settings were shown to yield high accuracy results in phonon-related properties compared to experimental measurements in crystalline naphthalene[14].

Table S 4: System-specifically chosen sampling meshes of (Γ-centred) electronic wave vectors, ***k****. Note that the order of integer sampling points along the reciprocal lattice vectors is given for the definitions of the unit cells as deposited in the CSD (see above).*

System	(Γ-centred) *k*-mesh
1A-o	2×2×1
1A	3×3×2
2A	2×3×2
3A	2×3×2
4A	2×2×1
5A	2×2×1
5A-II	2×2×1

The headers of the PAW-pseudopotentials that we used for the DFT calculations (for carbon and hydrogen atoms) are listed in *Table S 5*.

Table S 5: Headers of the used (standard) pseudopotentials for all atomic species.

**Atomic species**	**Pseudopotential header**
C	PAW_PBE C 08Apr2002
H	PAW_PBE H 15Jun2001

S4.2 Geometry and unit cell optimisations

The atomic positions and the lattice parameters were optimized to residual forces below 1 meV/Å employing the conjugate gradient algorithm within the following procedure: the independent lattice parameters and the atomic positions were optimised at a few fixed unit-cell volumes (using the *VASP* optimisation keyword `ISIF=4`). This procedure avoids Pulay stresses[15] to a large extent.[11] The optimum unit-cell volume was subsequently determined by fitting total energies of those structures with optimized lattice constants and atomic positions but fixed unit-cell volumes to a Rose-Vinet equation of state[16]. Finally, a last optimization was carried out at the optimal volume extracted from the fitted equation of state to obtain the final atomic positions and lattice parameters.

S4.3 Phonon calculations

Regarding the phonon calculations using the *Phonopy* code,[17] a displacement distance of 0.01 Å was used to displace the atoms in supercells of system-specifically adjusted extents (see Table S 6) which were chosen based on thorough convergence tests for 2A.[14] For band structure plots, the reciprocal space between each pair of high-symmetry points was sampled by 200 intermediate points, while for quantities that rely on a homogeneous sampling of the entire first Brillouin zone (density of states/group velocities, heat capacity) system-specific meshes of phonon wave vectors, $\boldsymbol{q}$, were used (see Table S 6). For the group velocities, the meshes were shifted such that they do not include the centre of the first Brillouin zones, Γ. Table S 6 shows two choices of $\boldsymbol{q}$-meshes

which sample the respective first Brillouin zones with different densities. The “dense” meshes (with a maximum integer of 15) were used for the (computationally more demanding) two-dimensional densities of frequencies and group velocities, while the “highly dense” meshes (with a maximum integer of 30) were used for densities of states (DOSs), densities of group velocities (DOGVs) and for the calculation of the heat capacity, C_V.

Table S 6: System-specifically chosen supercell sizes. Note that the order of integer replication factors along the lattice vectors as well as the order of integer sampling points along the reciprocal lattice vectors are given for the definitions of the unit cells as deposited in the CSD (see above).

System	Supercell size	q-meshes (dense)	q-mesh (highly dense)
1A-o	2×2×2	15×14×11	30×28×22
1A	3×3×2	15×14×11	30×28×22
2A	2×3×2	13×15×12	27×30×25
3A	2×3×2	13×15×10	26×30×20
4A	2×2×2	15×11×7	30×23×14
5A	2×2×2	12×15×6	25×30×12
5A-II	2×2×2	15×13×7	30×25×13

S4.4 Densities of states per frequency and per group velocity

Based on the uniformly sampled group velocities and frequencies, a two-dimensional density of states, g, per frequency, ω, and per group velocities, v_g, was calculated using the following definition:

$$g(\omega, v_g) = \frac{1}{3N}\frac{1}{N_q}\sum_{\lambda}\delta(\omega - \omega_\lambda)\,\delta(v_g - v_{g,\lambda}) \qquad \text{(S1).}$$

Here, $3N$ is the number of bands, N_q the number of wave vectors used in the sum over the joint index λ containing the band index and the wave vector. The delta distributions in Equation (S1) were replaced by Lorentzian functions of finite widths of σ_{vg} = 0.02 THzÅ (=0.002 kms^{-1}) for group velocities and σ_ω = 0.05 THz for frequencies (with x representing either group velocities or frequencies):

$$\delta(x - x_\lambda) \rightarrow \frac{1}{\pi} \frac{\sigma_x}{(x - x_\lambda)^2 + \sigma_x^2} \tag{S2}$$

In other words, the finite broadening parameter, σ_x, corresponds to the half width at half maximum of the artificially broadened peaks.

From the density g in frequency-group velocity space, one can, in principle, directly calculate the density of states (DOS; density of states per frequency) and the density of states per group velocity (DOGV) by integrating Equation (S1) over the (continuous) group velocity or frequency variable, respectively:

$$\frac{DOS(\omega)}{3N} = \int dv_g \;\; g(\omega, v_g) = \frac{1}{3N} \frac{1}{N_q} \sum_\lambda \delta(\omega - \omega_\lambda) \tag{S3}$$

$$\frac{DOGV(v_g)}{3N} = \int d\omega \;\; g(\omega, v_g) = \frac{1}{3N} \frac{1}{N_q} \sum_\lambda \delta(v_g - v_{g,\lambda}) \tag{S4}$$

Although these integrations could be carried out numerically to obtain the DOS and the DOGV from g, these two quantities were calculated separately using denser meshes of (phonon) wave vectors, $\boldsymbol{q}$, using Equation (S3) and (S4) directly. The reason why less dense meshes were used for the computation of the density g in the first place, was a notable decrease in computational efficiency with the density of the $\boldsymbol{q}$-mesh: for denser meshes, no visually perceivable changes in g were observed any more, while the computational time (due to fact that large two-dimensional arrays had to be stored and changed) significantly increased.

For the sake of completeness, the following plots show the DOS, the DOGV and the two-dimensional densities, g, for all studied molecular crystals.

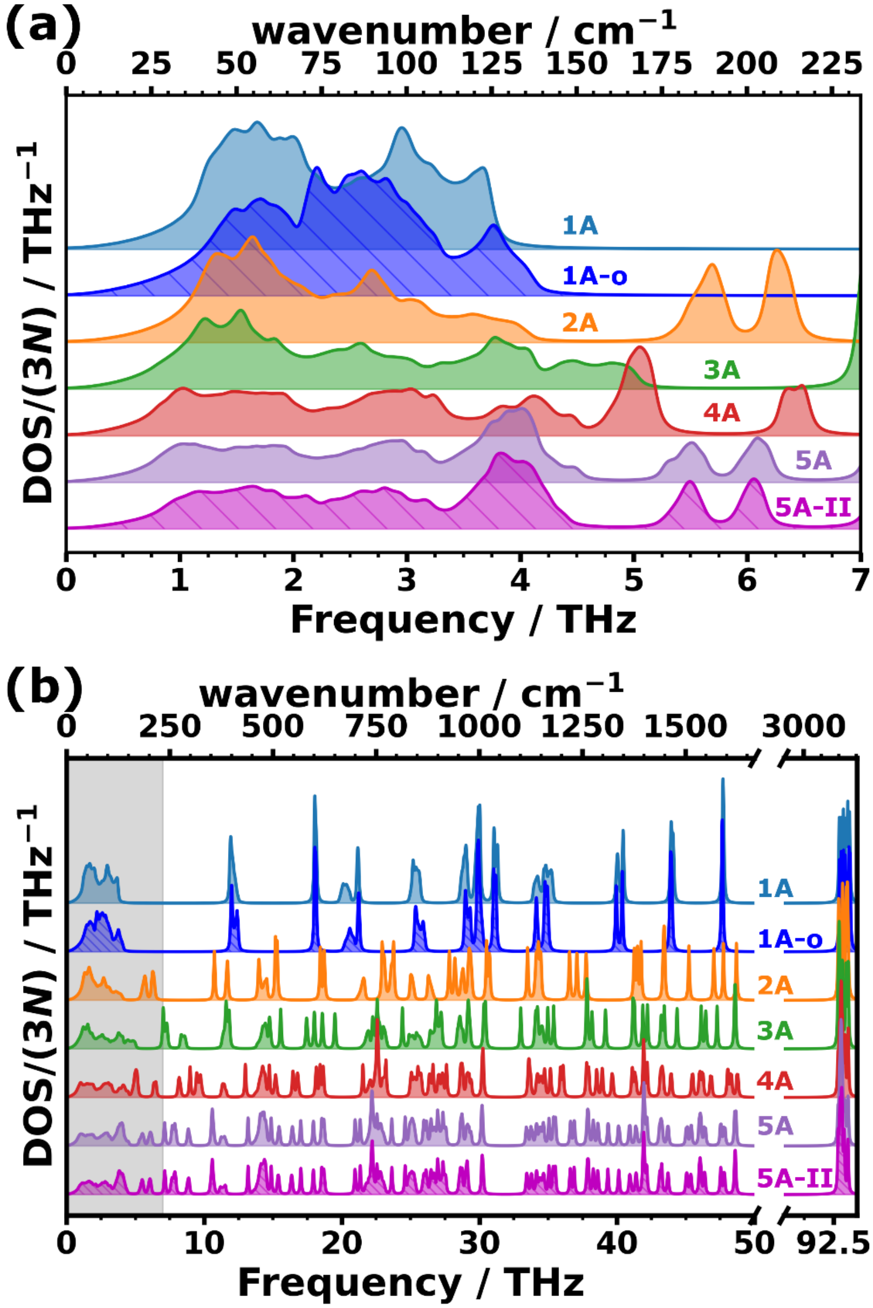

Figure S 6: Phonon DOSs (normalised by the number of bands, 3N) of the studied organic semiconductor crystals (a) in the low-frequency region ≤ 7 THz and (b) in the entire spectral range. The low-frequency region is indicated in panel (b) by the grey shaded area. The hatched DOSs highlight those polymorphs that were not treated explicitly in the main text.

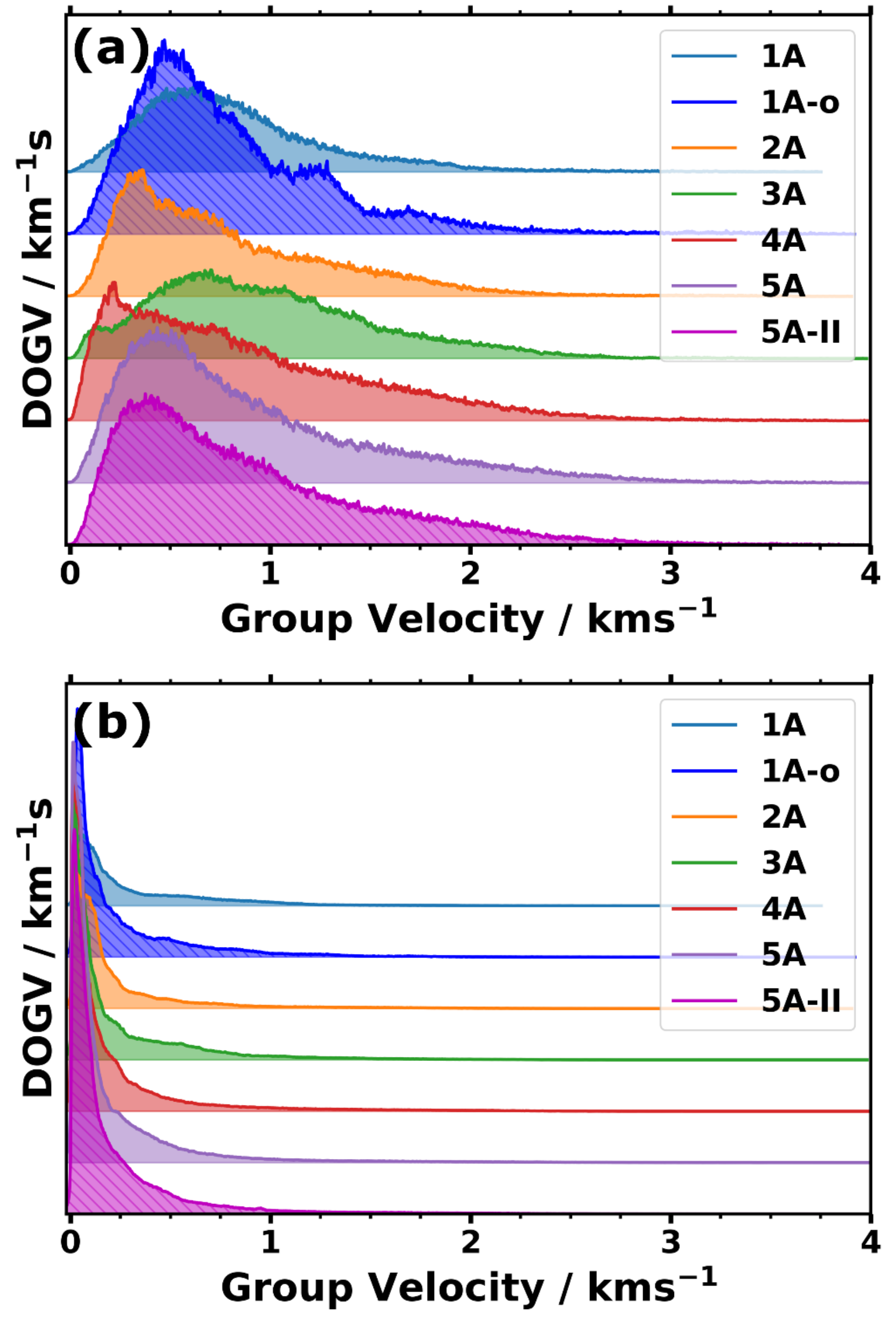

Figure S 7: Density of states per group velocity (DOGVs) of the studied organic semiconductor crystals (a) if only the modes in the low-frequency region ≤ 7 THz are considered and (b) if the entire spectral range is considered. The hatched DOGVs highlight those polymorphs that were not treated explicitly in the main text.

As far as the evolution of the group velocities with molecular length is concerned, one can see in the density of states per group velocities (DOGV) which has been restricted to phonons with frequencies ≤ 7 THz (see Figure S 7(a)) the various systems tend to peak at group velocities between ~0.25 and 0.75 kms^{-1}. Additionally, an odd-even effect seems to be visible in the low-frequency DOGV: while 2A and 4A show a pronounced (narrow) maximum al low group velocities, this maximum vanishes in 1A and 5A. Also in 3A it is considerably smaller, although it does not vanish. This means that 2A and 4A have a comparably large part of low-v_g bands ≤ 7 THz. The exact reason for this remains elusive, but it is likely the result of a complex superposition of strong shifts/weak shifts of bands, avoided crossings, unit-cell extents etc.

When considering all phonons (i.e., with no frequency restriction; see Figure S 7(b)), the DOGVs for low group velocities are drastically increased, which is a consequence of most intramolecular bands being rather flat due to a weak interaction between molecules for the respective motions. Although still, the DOGV for high group velocities slightly increases with the molecule length from 1A to 5A, the increasing number of comparably flat high-frequency intramolecular bands in the larger system causes the medians (maxima) of the DOGVs to shift considerably to lower frequencies. Here, a monotonic decrease of the median position of the all-phonon DOGVs is observed from 1A (~0.057 kms^{-1}) – with 1A-o already showing a sharp decrease to ~0.035 kms^{-1} - to 5A (~0.014 kms^{-1}).

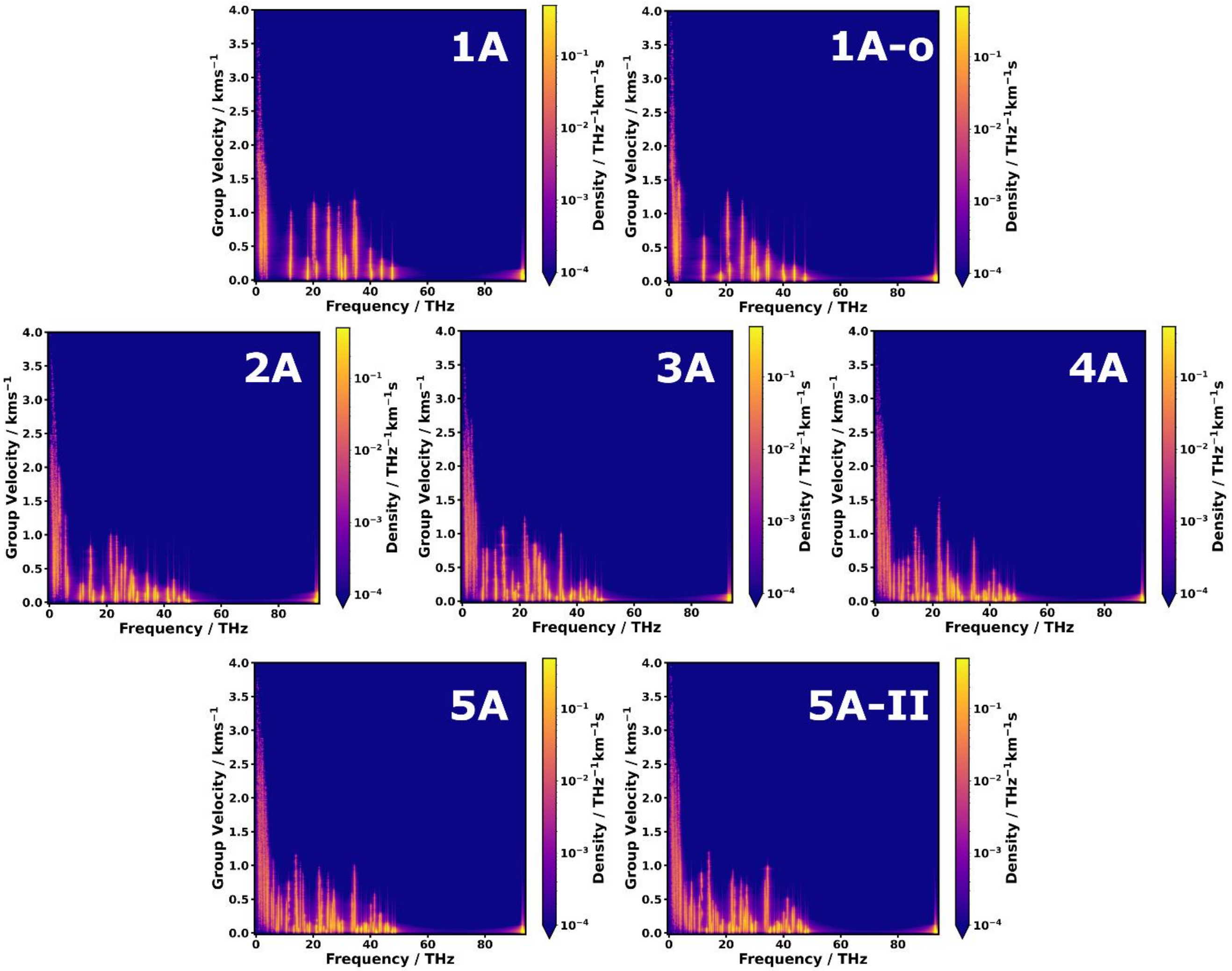

Figure S 8: Two-dimensional densities in group velocity-frequency-space, g (see Equation (S1)), for all studied organic semiconductor crystals. The density is plotted as the (logarithmic) colour scale.

S4.5 Participation ratios

The participation ratio (PR) of a phonon mode λ describes the degree of localization of the atomic motion associated with the phonon mode.[18–21] Therefore, the PR is a convenient measure to quantify the extent of moving atoms in the unit cell. According to its definition,

$$PR_\lambda = \frac{\left(\sum_{i=1}^{N}\sum_{\alpha=1}^{3} m_i^{-1}\left|\boldsymbol{e}_{i,\lambda}^{\alpha}\right|^2\right)^2}{N\left(\sum_{i=1}^{N}\sum_{\alpha=1}^{3} m_i^{-2}\left|\boldsymbol{e}_{i,\lambda}^{\alpha}\right|^4\right)} \tag{S5}$$

the PR depends on the atomic masses, m_i, of the N atoms in the unit cell and on the αth Cartesian component of the phonon (polarisation) eigenvectors, $\boldsymbol{e}^{\alpha}_{\iota,\lambda}$. To calculate the PR of a mode, one must carry out the above summation over Cartesian directions, α, and atoms, i.

One can easily verify that the PR ranges between 1 (if all atoms move with the same amplitude) and $1/N$ (if only one atom in the unit cell moves).

S5. Animations of phonon modes

S5.1 Γ-phonons

To facilitate the association of the provided Γ-phonon animations with the discussed vibrations, the following tables list the frequencies and types of modes that the video files show.

Table S 7: Animations of Γ-phonons of 1A. The relative phases between the motions of the two molecules per unit cell are abbreviated as IP (in-phase) and AP (antiphase).

Frequency / THz	Type of Mode	File Name
1.21	RRIMM $\boldsymbol{a_1}$/normal IP	mode04_01d21THz.mp4
1.89	RRIMM $\boldsymbol{a_1}$/normal AP	mode05_01d89THz.mp4
2.10	TRIMM $\boldsymbol{a_3}$/long	mode06_02d10THz.mp4
2.62	TRIMM $\boldsymbol{a_2}$/short	mode07_02d62THz.mp4
2.93	RRIMM $\boldsymbol{a_2}$/short IP	mode08_02d93THz.mp4
3.13	RRIMM $\boldsymbol{a_2}$/short AP	mode09_03d13THz.mp4
3.14	TRIMM $\boldsymbol{a_1}$/normal	mode10_03d14THz.mp4
3.67	RRIMM $\boldsymbol{a_3}$/long AP	mode11_03d67THz.mp4
3.76	RRIMM $\boldsymbol{a_3}$/long IP	mode12_03d76THz.mp4

Table S 8: Animations of Γ-phonons of 2A. The relative phases between the motions of the two molecules per unit cell are abbreviated as IP (in-phase) and AP (antiphase).

Frequency / THz	Type of Mode	File Name
1.57	RRIMM $\boldsymbol{a_1}$/normal IP	mode04_01d57THz.mp4
1.66	TRIMM $\boldsymbol{a_3}$/long	mode05_01d66THz.mp4
1.85	RRIMM $\boldsymbol{a_1}$/normal AP	mode06_01d85THz.mp4

2.27	TRIMM $\boldsymbol{a_2}$/short	mode07_02d27THz.mp4
2.43	RRIMM $\boldsymbol{a_2}$/short AP	mode08_02d43THz.mp4
2.60	RRIMM $\boldsymbol{a_2}$/short IP	mode09_02d60THz.mp4
3.20	TRIMM $\boldsymbol{a_1}$/dist.	mode10_03d20THz.mp4
3.56	RRIMM $\boldsymbol{a_3}$/long AP	mode11_03d56THz.mp4
4.11	RRIMM $\boldsymbol{a_3}$/long IP	mode12_04d11THz.mp4
5.27	Bending IP	mode13_05d27THz.mp4
5.78	Bending AP	mode14_05d78THz.mp4
6.37	Torsion IP	mode15_06d37THz.mp4
6.41	Torsion AP	mode16_06d41THz.mp4

Table S 9: Animations of Γ-phonons of 3A. The relative phases between the motions of the two molecules per unit cell are abbreviated as IP (in-phase) and AP (antiphase).

**Frequency / THz**	**Type of Mode**	**File Name**
1.31	RRIMM $\boldsymbol{a_1}$/normal AP	mode04_01d31THz.mp4
1.39	TRIMM $\boldsymbol{a_3}$/long	mode05_01d39THz.mp4
1.53	RRIMM $\boldsymbol{a_1}$/normal IP	mode06_01d53THz.mp4
2.08	RRIMM $\boldsymbol{a_2}$/short AP	mode07_02d08THz.mp4
2.10	TRIMM $\boldsymbol{a_2}$/short	mode08_02d10THz.mp4
2.39	RRIMM $\boldsymbol{a_2}$/short IP	mode09_02d39THz.mp4
3.15	TRIMM $\boldsymbol{a_1}$/normal	mode10_03d15THz.mp4
3.21	Bending IP	mode11_03d21THz.mp4
3.77	Bending AP	mode12_03d77THz.mp4
3.86	RRIMM $\boldsymbol{a_3}$/long AP	mode13_03d86THz.mp4
4.09	RRIM $\boldsymbol{a_3}$/long IP	mode14_04d09THz.mp4
4.96	Torsion IP	mode15_04d96THz.mp4
5.01	Torsion AP	mode16_05d01THz.mp4

Table S 10: Animations of Γ-phonons of 4A. The relative phases between the motions of the two molecules per unit cell are abbreviated as IP (in-phase) and AP (antiphase).

Frequency / THz	Type of Mode	File Name
1.09	TRIMM $\boldsymbol{a_3}$/long	mode04_01d09THz.mp4
1.42	RRIMM $\boldsymbol{a_1}$/normal IP	mode05_01d42THz.mp4
1.54	RRIMM $\boldsymbol{a_1}$/normal AP	mode06_01d54THz.mp4
1.93	RRIMM $\boldsymbol{a_2}$/short IP	mode07_01d93THz.mp4
2.11	Bending IP	mode08_02d11THz.mp4
2.15	TRIMM $\boldsymbol{a_2}$/short	mode09_02d15THz.mp4
2.78	RRIMM $\boldsymbol{a_2}$/short AP	mode10_02d78THz.mp4
3.15	TRIMM $\boldsymbol{a_1}$/normal	mode11_03d15THz.mp4
3.26	Bending AP	mode12_03d26THz.mp4
3.74	RRIMM $\boldsymbol{a_3}$/long AP	mode13_03d74THz.mp4
4.12	RRIMM $\boldsymbol{a_3}$/long IP	mode14_04d12THz.mp4
4.23	Torsion IP	mode15_04d23THz.mp4
4.51	Torsion AP	mode16_04d51THz.mp4

Table S 11: Animations of Γ-phonons of 5A. The relative phases between the motions of the two molecules per unit cell are abbreviated as IP (in-phase) and AP (antiphase).

Frequency / THz	Type of Mode	File Name
1.15	TRIMM $\boldsymbol{a_3}$/long	mode04_01d15THz.mp4
1.25	RRIMM $\boldsymbol{a_1}$/normal IP	mode05_01d25THz.mp4
1.51	RRIMM $\boldsymbol{a_1}$/normal AP	mode06_01d51THz.mp4
1.75	Bending IP	mode07_01d75THz.mp4
1.85	RRIMM $\boldsymbol{a_2}$/short IP	mode08_01d85THz.mp4
2.19	TRIMM $\boldsymbol{a_2}$/short	mode09_02d19THz.mp4
2.85	RRIMM $\boldsymbol{a_2}$/short AP	mode10_02d85THz.mp4
2.96	Bending AP	mode11_02d96THz.mp4
3.22	TRIMM $\boldsymbol{a_1}$/normal	mode12_03d22THz.mp4
3.65	RRIMM $\boldsymbol{a_3}$/long AP	mode13_03d65THz.mp4
3.77	In-Plane Bending IP	mode14_03d77THz.mp4

3.85	In-Plane Bending AP	mode15_03d85THz.mp4
4.05	Second-Order Bending IP	mode16_04d05THz.mp4
4.11	Torsion AP	mode17_04d11THz.mp4
4.12	Second-Order Bending AP	mode18_04d12THz.mp4
4.24	RRIMM $\boldsymbol{a_3}$/long IP	mode19_04d24THz.mp4
4.51	Torsion IP	mode20_04d51THz.mp4
5.50	Second-Order Torsion AP	mode21_05d50THz.mp4
5.64	Second-Order Torsion IP	mode22_05d64THz.mp4

Table S 12: Animations of Γ-phonons of 5A-II. The relative phases between the motions of the two molecules per unit cell are abbreviated as IP (in-phase) and AP (antiphase).

Frequency / THz	Type of Mode	File Name
1.18	RRIMM $\boldsymbol{a_2}$/short IP	mode04_01d18THz.mp4
1.31	TRIMM $\boldsymbol{a_3}$/long	mode05_01d31THz.mp4
1.72	RRIMM $\boldsymbol{a_1}$/normal IP	mode06_01d72THz.mp4
1.78	Bending IP	mode07_01d78THz.mp4
2.21	TRIMM $\boldsymbol{a_2}$/short	mode08_02d21THz.mp4
2.33	RRIMM $\boldsymbol{a_1}$/normal AP	mode09_02d33THz.mp4
2.84	Bending AP	mode10_02d84THz.mp4
2.84	RRIMM $\boldsymbol{a_2}$/short AP	mode11_02d84THz.mp4
3.22	TRIMM $\boldsymbol{a_1}$/normal	mode12_03d22THz.mp4
3.74	In-Plane Bending IP	mode13_03d74THz.mp4
3.75	Second-Order Bending IP	mode14_03d75THz.mp4
3.79	In-Plane Bending AP	mode15_03d79THz.mp4
3.90	RRIMM $\boldsymbol{a_3}$/long AP	mode16_03d90THz.mp4
3.96	Torsion AP	mode17_03d96THz.mp4
4.12	Second-Order Bending AP	mode18_04d12THz.mp4
4.24	RRIMM $\boldsymbol{a_3}$/long IP	mode19_04d24THz.mp4
4.40	Torsion IP	mode20_04d40THz.mp4

S5.2 Acoustic phonons close to Γ

In addition to the animations of Γ-phonons, also the acoustic phonons in selected directions were animated to gain some insight into the atomic displacements of the associated motions. The atomic geometries displaced along the phonon eigendisplacements (at $\boldsymbol{q} \neq 0$) were generated in a way analogous to the implementation in *Phonopy*.[17] The so-created displaced geometries were subsequently rendered using the *Ovito* software[22] and saved as files "TA1.mp4", "TA2.mp4", and "LA.mp4" (for the first transverse, the second transverse, and the longitudinal acoustic modes) in the respective subfolders according to the directions of the wave vectors (see Table S 13).

Table S 13: Coordinates of the wave vectors in 5A for which the acoustic modes were animated. Here, the vector norm of the wave vector was held at a constant value corresponding to the radius of the spheres around Γ, at which the sound velocities are calculated: for 5A, the chosen length of the wave vector is $\delta q = 4.42\ 10^{-3} Å^{-1}$. Additionally, to facilitate the visual recognition, one set of animations has been created with keeping the directions of the wave vectors constant but increasing its magnitude to $10\delta q$. The unit vectors of various selected directions are listed below. The direction $\boldsymbol{a}_3$ corresponds to the direction along the lattice vector $\boldsymbol{a}_3$, while $\boldsymbol{a}_1$' and $\boldsymbol{a}_2$' are the projections of $\boldsymbol{a}_1$ and $\boldsymbol{a}_2$ into the plane orthogonal to $\boldsymbol{a}_3$. The directions $\boldsymbol{b}_i$ correspond to the directions of the reciprocal lattice vectors. In the last column, the more common way of defining the wave vector in terms of the reciprocal lattice vectors, $\boldsymbol{b}_i$, is shown, where all shown wave vectors have the magnitude δq.

direction	Cartesian unit vectors	Wave vector (in units of reciprocal lattice vectors)
$\boldsymbol{a}_3$	[-0.3860, -0.1920, 0.9023]	[-0.00213, -0.00098, 0.01113]
$\boldsymbol{a}_1$'	[0.9225, -0.0743, 0.3788]	[0.00500, -0.00002, 0.00000]
$\boldsymbol{a}_2$'	[-0.0111, 0.9790, 0.2036]	[-0.00003, 0.00415, 0.00000]
$\boldsymbol{b}_1$	[0.9224, -0.0686, 0.3800]	[0.00500,0.00000,0.00000]
$\boldsymbol{b}_2$	[-0.0057, 0.9786, 0.2058]	[0.00000, 0.00415,0.00000]
$\boldsymbol{b}_3$	[0.0051, 0.0096, 0.9999]	[0.00000,0.00000,0.01000]

S6. Phonon band structures and first Brillouin zones

The following figures show the low-frequency phonon band structures of all studied molecular crystals along with their first Brillouin zones. There, also the high-symmetry points are displayed

explicitly to facilitate the recognition of the directions of the corresponding wave vectors with respect to the (reciprocal) lattice.

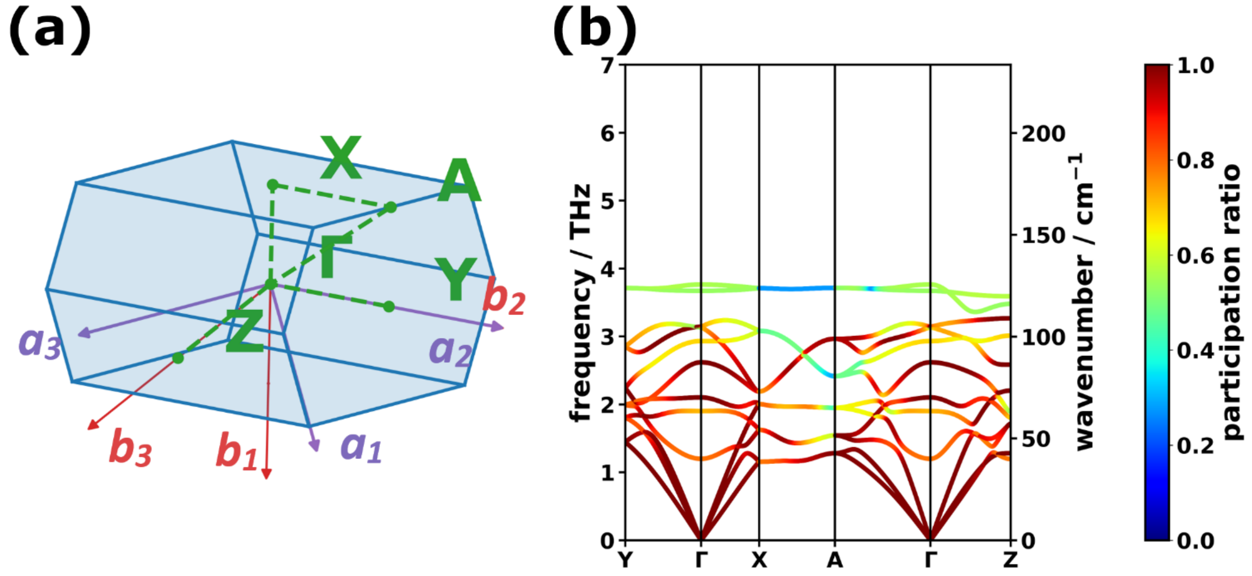

Figure S 9: (a) First Brillouin zone of the reordered lattice (see Section S3) of monoclinic benzene (1A) with the high-symmetry points, between which the phonon band structure in panel (b) is shown. The reciprocal, $\mathbf{b}_i$, and real-space lattice vectors, $\mathbf{a}_i$, are displayed as red and purple arrows, respectively. The bands are coloured according to their participation ratios (see Section S4.5).

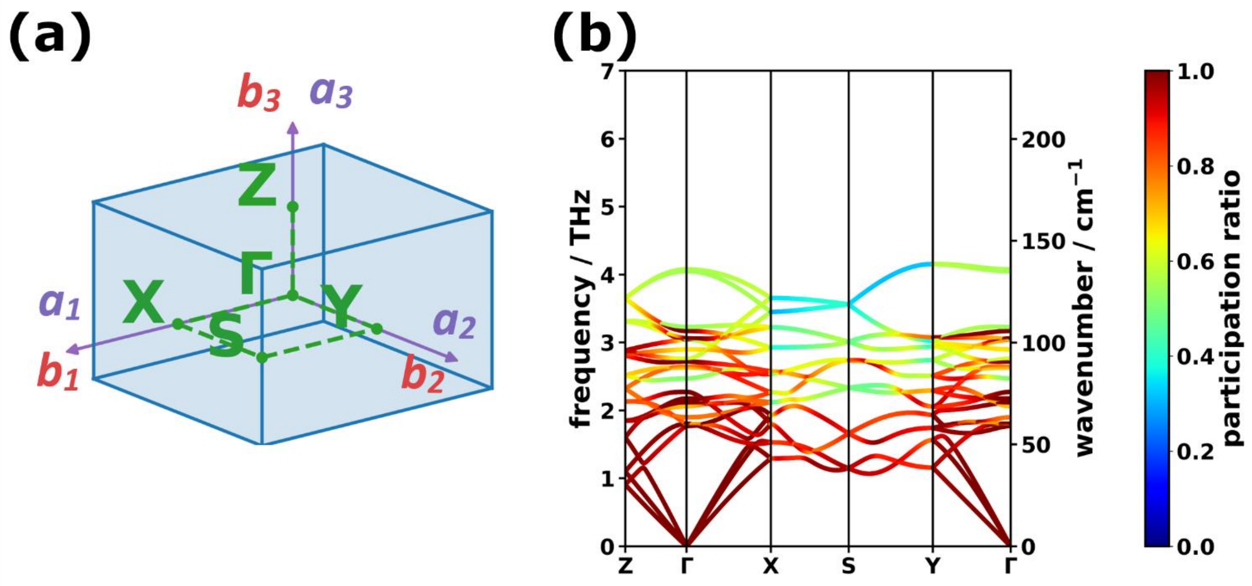

Figure S 10: (a) First Brillouin zone of orthorhombic benzene (1A-o) with the high-symmetry points, between which the phonon band structure in panel (b) is shown. The reciprocal, $\mathbf{b}_i$, and real-space lattice vectors, $\mathbf{a}_i$, are displayed as red and purple arrows, respectively. The bands are coloured according to their participation ratios (see Section S4.5).

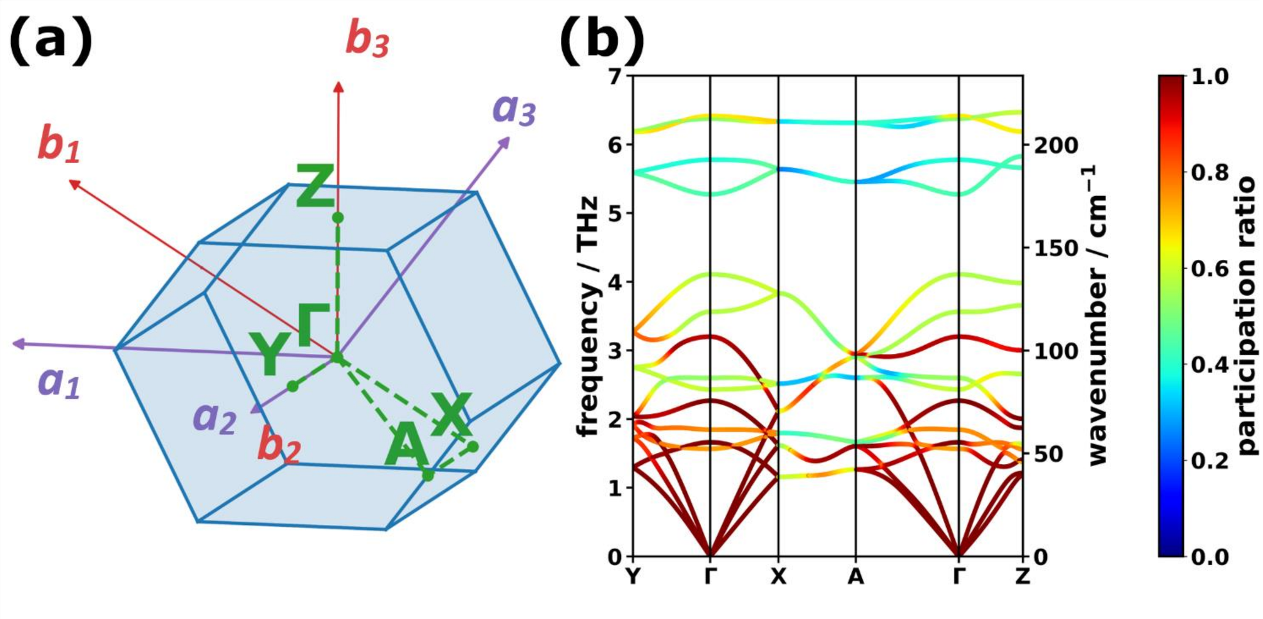

*Figure S 11: (a) First Brillouin zone of naphthalene (2A) with the high-symmetry points, between which the phonon band structure in panel (b) is shown. The reciprocal, **b_i**, and real-space lattice vectors, **a_i**, are displayed as red and purple arrows, respectively. The bands are coloured according to their participation ratios (see Section S4.5).*

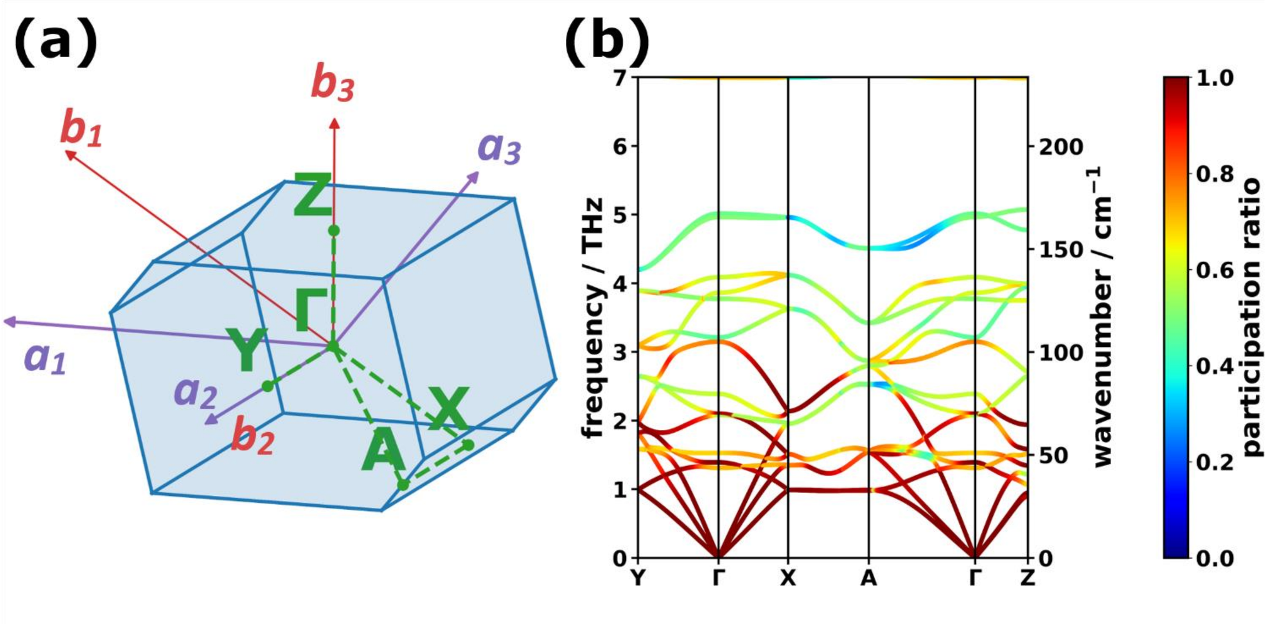

*Figure S 12: (a) First Brillouin zone of anthracene (3A) with the high-symmetry points, between which the phonon band structure in panel (b) is shown. The reciprocal, **b_i**, and real-space lattice vectors, **a_i**, are displayed as red and purple arrows, respectively. The bands are coloured according to their participation ratios (see Section S4.5).*

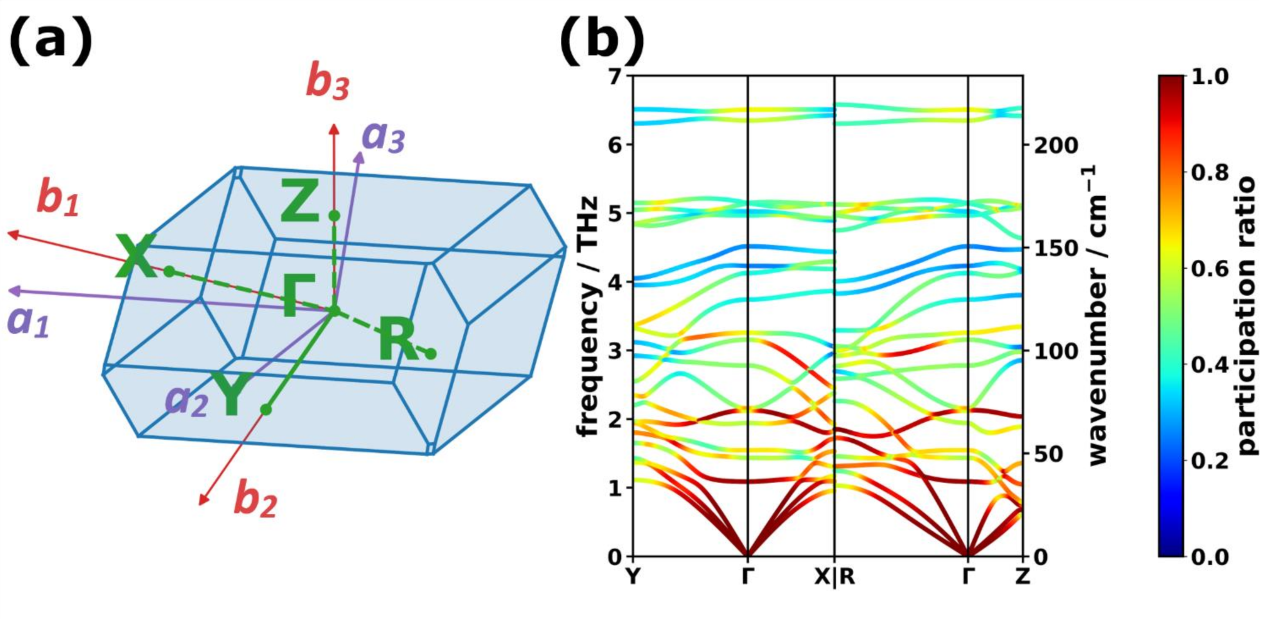

Figure S 13: (a) First Brillouin zone of the reordered lattice (see Section S3) of tetracene (4A) with the high-symmetry points, between which the phonon band structure in panel (b) is shown. The reciprocal, ***b****$_i$, and real-space lattice vectors,* ***a****$_i$, are displayed as red and purple arrows, respectively. The bands are coloured according to their participation ratios (see Section S4.5).*

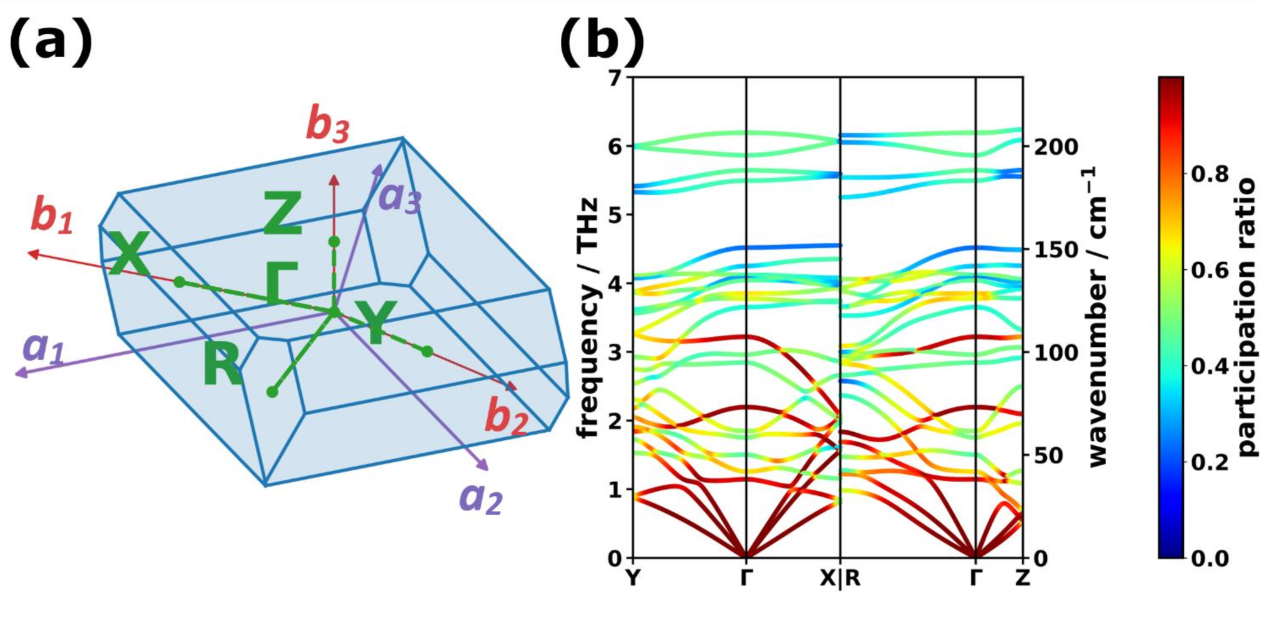

Figure S 14: (a) First Brillouin zone of pentacene polymorph I (5A) with the high-symmetry points, between which the phonon band structure in panel (b) is shown. The reciprocal, ***b****$_i$, and real-space lattice vectors,* ***a****$_i$, are displayed as red and purple arrows, respectively. The bands are coloured according to their participation ratios (see Section S4.5).*

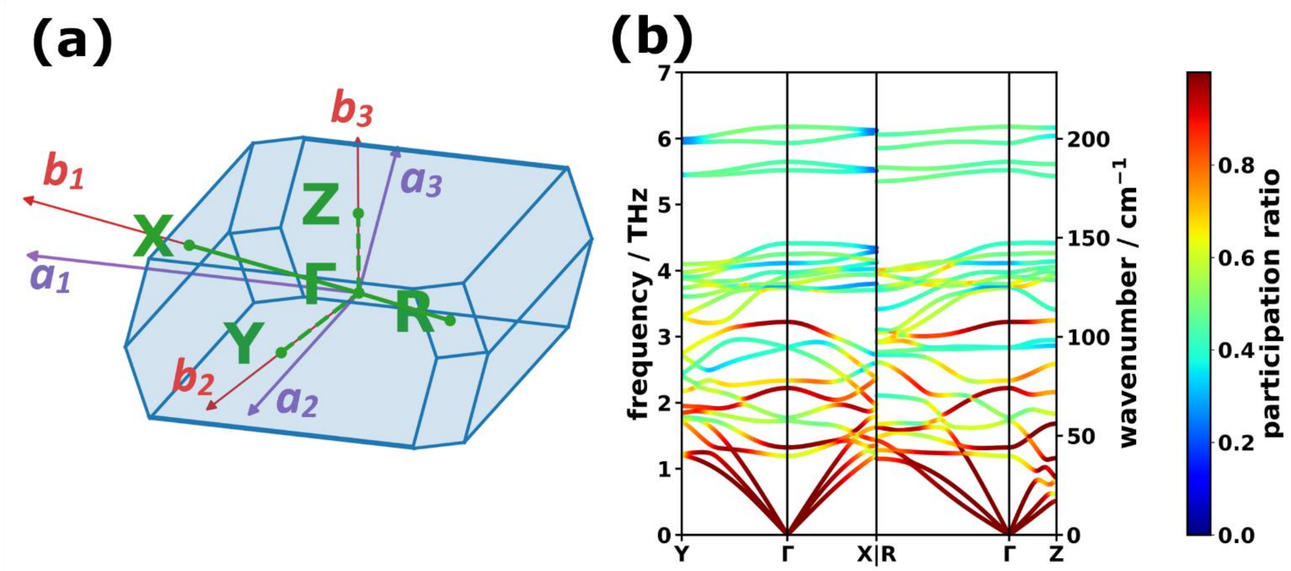

Figure S 15: (a) First Brillouin zone of the reordered lattice (see Section S3) of pentacene polymorph II (5A-II) with the high-symmetry points, between which the phonon band structure in panel (b) is shown. The reciprocal, $\mathbf{b}_i$, and real-space lattice vectors, $\boldsymbol{a}_i$, are displayed as red and purple arrows, respectively. The bands are coloured according to their participation ratios (see Section S4.5).

S7. Details on the classical models to describe the evolution of Γ-frequencies

S7.1 Intramolecular bending model

The classical analogue used to model the intramolecular bending frequencies in the aces is the bending bar. For a bar of length L, with mass density ρ, cross section area A, flexural rigidity EI (containing Young's modulus, E, and the second moment of area of the cross section, I), the (angular) eigenfrequencies, ω_m, of the transverse vibrations of the bar (labelled by the index m), are given by the following equation:[23]

$$\omega_m = \frac{\mu_m^2}{L^2}\sqrt{\frac{EI}{\rho A}} \tag{S6}$$

In addition to all these geometric and material-specific quantities, a dimensionless variable, μ_m, enters Equation (S6), which is only determined by the boundary conditions of the bar, i.e., the kind and position of the supports.[23] As most of the geometric and material-specific parameters in Equation (S6) would not allow a reasonable determination for the given (non-continuous) case of

bending molecules, the model is used to estimate frequency ratios rather than absolute frequencies. Here, we assume that A, ρ, and EI stay constant between the system and that the only parameter that varies is the "beam length", L. The remaining problem is to define the beam length for the bending molecules. In the simplest case of a bending bar, the transverse bending eigenmodes show nodes (i.e., positions of no transverse displacement) at the positions of the supports. That means, that a reasonable way to define the beam length in the molecules is to find those nodes and define the lengths between them as the "effective length", L_{eff}. An analysis of the bending vibrations (at Γ) reveals that this effective length approximately obeys the following empirically found equation:

$$L_{eff,n} = d_{ring} \cdot [n - (n-1)s] \tag{S7}$$

Here, d_{ring} is the (short) diameter of the hexagonal ring of benzene and n is an integer ranging from 1 (benzene, 1A) to 5 (pentacene, 5A) and, thus, counts the number of rings in the molecule. Additionally, a parameter, s, enters the above equation to account for the observation that the ratio of L_{eff} and the total molecular length decreases for longer acenes. For the bending modes, s=0.5 is used, whereas for the intramolecular in-plane bending modes (for which the same model based on a bending beam is employed), s=0.7 was found suitable to approximate L_{eff} in the atomic displacement patters and, thus, the associated frequencies.

Once L_{eff} is known, the following equation can be used to estimate the frequency ratios relative to a reference system:

$$\frac{\omega_n}{\omega_{ref}} = \frac{L_{eff,ref}^2}{L_{eff,n}^2} \tag{S8}$$

Here, we used naphthalene (2A) as a reference for the mode, as this molecule is the simplest case which has a defined long molecular axis and shows no phonon hybridisation effects.

S7.2 Intramolecular torsion model

Similarly to the case of bending molecules introduced above, we employed a simple model of a torsional oscillator – with a torsional stiffness, k_{tor}, and the torsional moment of inertia, I_{tor} – to describe the frequency evolution as a function of the number of rings, n:

$$\omega_n^2 = \frac{k_{tor,n}}{I_{tor,n}} \Rightarrow \frac{\omega_n^2}{\omega_{ref}^2} = \frac{k_{tor,n}}{k_{tor,ref}} \frac{I_{tor,ref}}{I_{tor,n}} \tag{S9}$$

As a next step, the two ingredients, k_{tot} and I_{tor}, should be discussed. To this end, it is vital to understand how the intramolecular torsional mode distort the molecular geometries. In those

modes, one can clearly observe a nodal line, which corresponds to the *y*-axis as shown in Figure S 16. All atoms above the *y*-axis (i.e. all atoms with $z > 0$) undergo torsional motion around the *z*-axis in one direction as one largely rigid unit, while all the atoms below the *y*-axis ($z < 0$) undergo a torsion in the other direction (as one largely rigid unit).

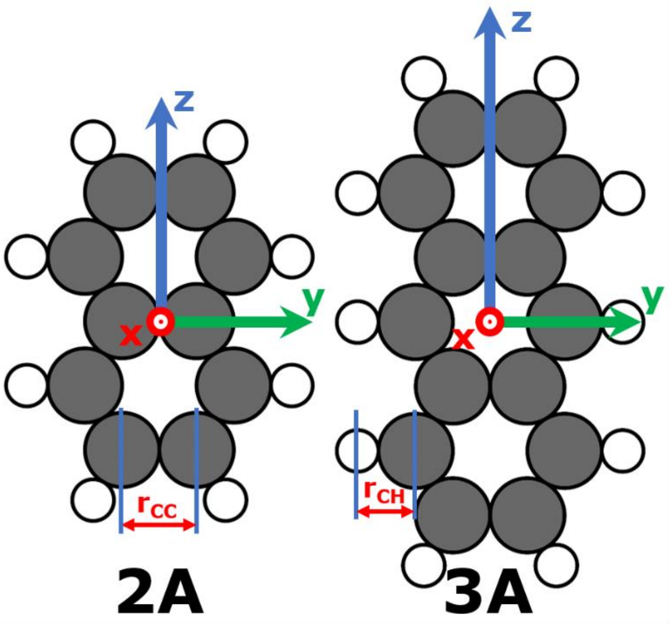

Figure S 16: Definition of the molecular Cartesian coordinate system (x,y,z), with respect to which the molecular moments of inertia were calculated. The distance between C and C, r_{CC}, and between C and H atoms, r_{CH}, are indicated by red double arrows. For the numeric calculations, these distances were set to constant values (1.396933 Å and 1.089885 Å), obtained from the average nearest-neighbour C-C and C-H distances in the crystal structure of monoclinic benzene (1A). Atomic colour coding: C…grey, H…white.

Depending on whether the number of rings, *n*, of the molecule is even (n mod 2 = 0) or odd (n mod 2 = 1), different atoms must be excluded from the total moment of inertia around the z-axis, Θ_{zz} (this quantity is introduced in Equation (S13) in Section S7.3): for even *n*, two central carbon atoms do not contribute to I_{tor} because they lie on the nodal plane and do not move, while for odd *n*, two carbons and to hydrogens must be excluded (see Figure S 16). For the sake of completeness, the analytic expression for I_{tor} used for the acenes with *n* rings is given in Equation (S10).

$$2\, I_{tor,n} = \Theta_{zz,n} - (n \bmod 2 - 1) \cdot 2m_C \left(\frac{r_{CC}}{2}\right)^2 - (n \bmod 2\,) \cdot\; 2(m_C r_{CC}^2 + m_H (r_{CC} + r_{CH})^2) \qquad \text{(S10)}$$

In addition to the total (rotational) moment of inertia around the *z*-axis, Θ_{zz} (as introduced in Equation (S13) below), I_{tor} depends on the atomic masses of the C and H atoms, m_C and m_H, and

the (average) nearest-neighbour C-C and C-H distance, r_{CC} and r_{CH}. Note that the numeric values (1.396933 Å and 1.089885 Å) for these distances were obtained from the average nearest-neighbour distances in benzene (1A) and were kept constant for all estimations of I_{tor} for the sake of simplicity.

Besides this odd-even effect in the calculation of I_{tor}, also the torsional stiffness, k_{tor}, is supposed to depend on the precise location of the nodal plane. Since the atoms with $y > 0$ and those with $y < 0$ undergo torsions as largely rigid units, the strongest local bond distortions are observed close to the nodal plane ($y = 0$). Therefore, we consider the local bond geometries in the proximity of the nodal plane as the restoring "springs" of this vibration. In case of odd-ringed acenes, this directly affects four C-C bonds, which are distorted from the energetically preferred planar (aromatic) arrangement. We consider this number of distorted bonds in the torsional stiffness as four times an arbitrary torsional reference stiffness, γ^0_{tor}. In case of even-ringed acenes, also the C-C bond in the centre of the molecule finds itself in an environment of non-planar bonds. Since this bond is "doubly unsatisfied", as the bonding environment on both sides is changing in opposite directions, this bond is counted twice such that k_{tor} equals six times γ^0_{tor} in this case. To sum up, the torsional stiffness, k_{tor}, is modelled to depend on the number of rings, n, in the following way:

$$k_{tor,n} = 2\gamma^0_{tor} \cdot (3 - n \bmod 2) \quad \text{(S11)}$$

S7.3 Rotational rigid intramolecular modes model

In analogy to the analysis of the translational rigid intermolecular modes (TRIMMs) presented in the main text, a similar analysis was applied also to the rotational rigid intermolecular modes (RRIMMs). For a rotational vibration, a suitable measure for the inertia of the oscillator is the appropriate moment of inertia, Θ_{ii}, which must be calculated for rotations around all axes separately (see below). If this quantity is known, the effective rotational molecular stiffness, K_{rot}, can subsequently be calculated from the (angular) frequencies:

$$\omega^2_{n,i} = \frac{K_{rot,n}}{\Theta_{ii}} \quad \text{(S12)}$$

In order to keep the model as simple as possible, the moments of inertia were calculated for rotations around the long and short molecular axis as well as around the axis normal through the π-planes of the molecule (corresponding to the *z*-, -*y*, and *x*-axes as shown in Figure S 16,

respectively). For the sake of completeness, the analytic expressions for those three moments of inertia as a function of the number of rings, n, are given below:

$$\Theta_{zz,n} = \left[m_C \frac{r_{CC}^2}{2} \cdot (5n+1) + m_H (r_{CC} + r_{CH})^2 \cdot (2n+1)\right] \tag{S13}$$

$$\Theta_{yy,n} = \left[3 m_C r_{CC}^2 \sum_{k=1}^{n} k^2 \; + \; 3 m_H \left((n r_{CC} + r_{CH})^2 + \; r_{CC}^2 \sum_{k=1-n \bmod 2}^{\frac{n-n \bmod 2}{2}} (2k - 1 + n \bmod 2)^2 \right)\right] \tag{S14}$$

$$\Theta_{xx,n} = \begin{cases} 6(m_C r_{CC}^2 + m_H (r_{CC} + r_{CH})^2) & \text{for } n = 1 \\ \left(\frac{41}{2} m_C r_{CC}^2 + 2 m_H (10 r_{CC}^2 + 11 r_{CC} r_{CH} + 4 r_{CH}^2)\right) & \text{for } n = 2 \\ \left(50\, m_C r_{CC}^2 + 2 m_H (23 r_{CC}^2 + 16 r_{CC} r_{CH} + 5 r_{CH}^2)\right) & \text{for } n = 3 \\ \left(\frac{201}{2} m_C r_{CC}^2 + m_H (87 r_{CC}^2 + 42 r_{CC} r_{CH} + 12 r_{CH}^2)\right) & \text{for } n = 4 \\ \left(178\, m_C r_{CC}^2 + 2 m_H (73 r_{CC}^2 + 26 r_{CC} r_{CH} + 7 r_{CH}^2)\right) & \text{for } n = 5 \end{cases} \tag{S15}$$

As already discussed in the main text, this calculation of the moments of inertia is somewhat problematic as the axes around which the rotations are observed do not always perfectly agree with the symmetry axes of the molecules, for which the expressions in Equation (S13) to (S15) hold. Nevertheless, using the numeric values obtained from the above equations still allows to draw several conclusions when analysing the resulting effective rotational force constants as a function of the associated moments of inertia. This analysis is shown in Figure S 17. Especially for the RRIMMs corresponding to rotations around the long molecular axes (see Figure S 17(a)), the data points lie quite nicely on fitted linear functions (through the origin). This is in agreement with the observation discussed in the main text that the frequencies of those kind of in-phase and antiphase RRIMMs are relatively independent of the molecule length. Only benzene shows a notable deviation from this trend in the in-phase RRIMM, suggesting lower rotational force constants than the longer acenes.

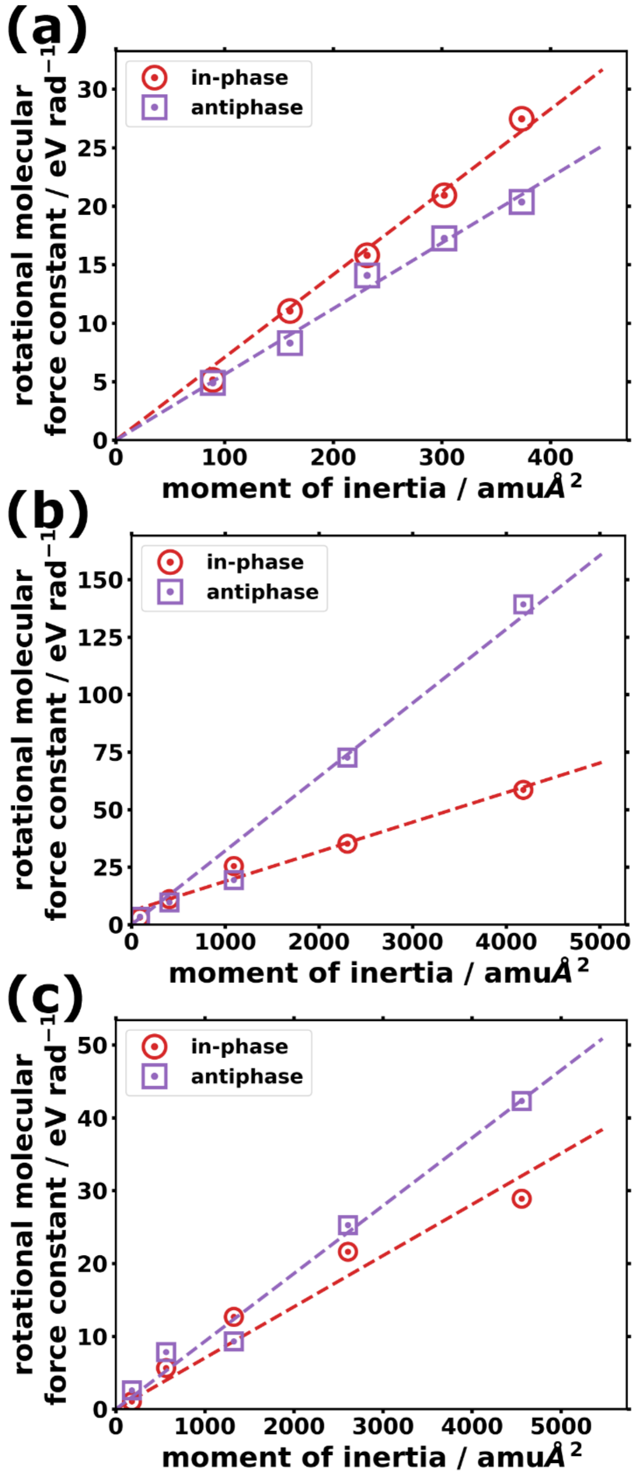

Figure S 17: Calculated effective intermolecular mode force constants for the three RIMMs (a) around the long molecular axes, (b) around the short molecular axes, and (c) around the axes normal to the π-planes in the studied molecular crystals as a function of the associated molecular moments of inertia. The dashed lines correspond to fitted linear functions through the origin for the RRIMMs with the exception of the in-phase RRIMM in panel (b), for which also the offset to the origin was fitted.

The evolution of the rotational force constants for RRIMMs corresponding (roughly) to rotations around the short molecular axes (see Figure S 17(b)) show much more unclear trends. Although for the antiphase RRIMM, a linear function through the origin seems to be suitable to describe the evolution (with antracene and naphthalene lying distinctly below the trend), for the in-phase RRIMM, a first-order polynomial (i.e., slope and offset) were fitted. In this case, a clear trend of decreasing frequency with the molecule length is observed (by a factor of ~1.6 in total from 1A to 5A). Therefore, the rotational force constants are not supposed to depend linearly on the moments of inertia (which would result in a constant frequency). Instead, due to the positive offset, b_0, and the slope, b_1, the frequencies are expected to decrease with $\sqrt{b_1 + b_0/\Theta_{yy}}$. However, direct connections to the molecular length are hampered by the fact that in contrast to Θ_{zz}, Θ_{yy} depends on the number of rings per molecule in a non-linear way.

Finally, for the last type of RRIMMs, again a more or less linear relationship between Θ_{xx} and the calculated rotational force constant can be observed (suggesting roughly constant frequencies); the deviations from the linear trends are, however, larger than in all other cases discussed so far. Still, it seems that the increase in the magnitude of the effective rotational force constants can keep up with the (superlinear) increase in the moment of inertia (approximately) associated with this kind of motion.

S8. Sound velocities (long-wavelength limit)

For the spatial distributions of sound velocities, the group velocities of the acoustic bands were calculated for a small sphere (with radius equalling 1 % of the length of the shortest reciprocal lattice vector in each system to guarantee comparability; see Table S 14) on a 360×180 mesh of azimuthal, ϕ, and polar angles, θ, respectively. For the sake of completeness, the average, maximum, minimum and standard deviation of the $TA_{(1,2)}$ and LA sound velocities on these reciprocal-space spheres are listed in Table S 14, and the deviations from the respective averages are plotted as projections onto unit spheres for all studied systems in the following.

Note that, since the sound velocities , were sampled at meshes in spherical coordinates, average values, $\langle v_p \rangle$, and standard deviations, $\mathrm{std}(v_p)$,were calculated in the following way (with p = $TA_1/TA_2/LA$)

$$\langle v_p \rangle = \frac{1}{4\pi}\int_0^{2\pi} d\phi \int_0^{\pi} d\theta \;\; v_p(\theta,\phi)\sin(\theta) \tag{S16}$$

$$\mathrm{std}(v_p) = \sqrt{\langle v_p^2 \rangle - \langle v_p \rangle^2} \tag{S17}$$

*Table S 14: **q**-radius, δq, of the spheres in reciprocal space, on the surface of which the sound velocities were calculated for the studied systems. Additionally, the average (Av.), minimum (Min.), maximum (Max.) and the standard deviation (Std.) of the respective group velocity (vector norms) on those spheres are listed.*

OSC	δq /	TA_1 mode / kms^{-1}				TA_2 mode / kms^{-1}				LA mode / kms^{-1}			
	10^{-3} Å^{-1}	Av.	Min.	Max.	Std.	Av.	Min.	Max.	Std.	Av.	Min.	Max.	Std.
1A-o	6.76	1.96	0.78	3.64	0.46	2.39	1.77	3.73	0.48	3.64	2.94	3.98	0.26
1A	8.67	1.81	1.45	2.64	0.15	2.36	1.81	3.27	0.37	3.54	2.85	3.86	0.21
2A	8.82	1.92	1.39	2.54	0.25	2.35	1.74	2.99	0.22	3.63	2.90	4.05	0.21
3A	6.94	1.76	1.17	2.57	0.32	2.31	1.42	2.93	0.28	3.56	2.95	4.16	0.30
4A	5.21	2.19	0.77	3.73	0.67	2.98	1.29	4.09	0.53	4.07	2.88	4.99	0.58
5A	4.42	2.47	0.64	4.55	0.79	3.17	1.22	4.63	0.65	4.30	2.77	5.33	0.71
5A-II	4.51	2.38	0.70	4.41	0.66	3.12	1.53	4.54	0.63	4.32	3.02	5.32	0.67

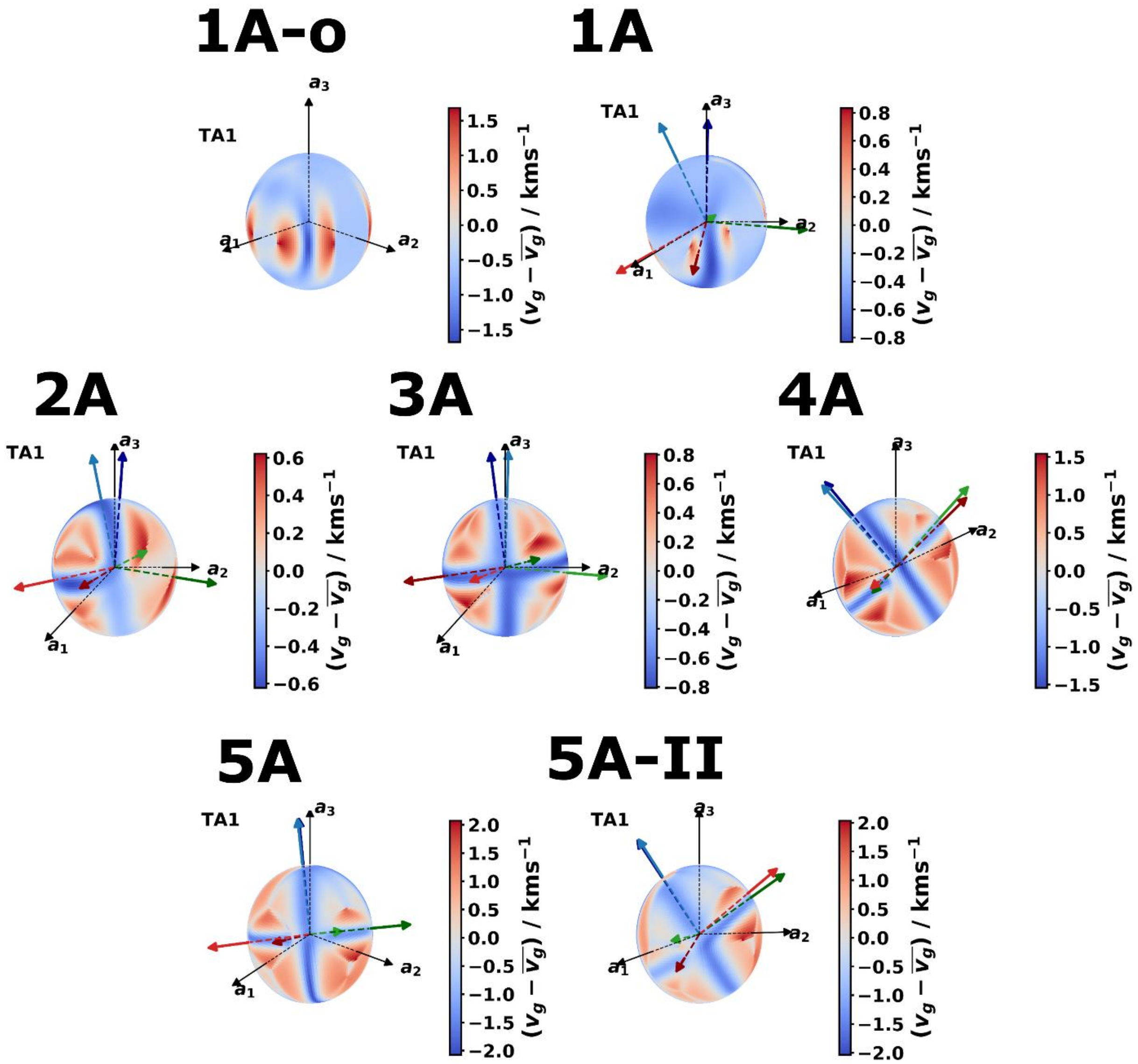

Figure S 18: Differences in the first (largely) transverse acoustic (TA_1) sound velocities and the corresponding mean values (see Table S 14) for all studied organic semiconductor crystals. The lattice vectors, $\boldsymbol{a}_1$, $\boldsymbol{a}_2$, and $\boldsymbol{a}_3$, are shown as black arrows. Additionally, the long molecular axes (blue arrows), the short molecular axes (red arrows) and the normal vectors to the π-planes (green arrows) of the two molecules per unit cell are indicated. The three arrows with a slightly darker shade belong to one molecule, while the lighter ones belong to the other. As 1A-o contains four molecules per unit cell, no additional molecular directions are shown to avoid perceptual confusion.

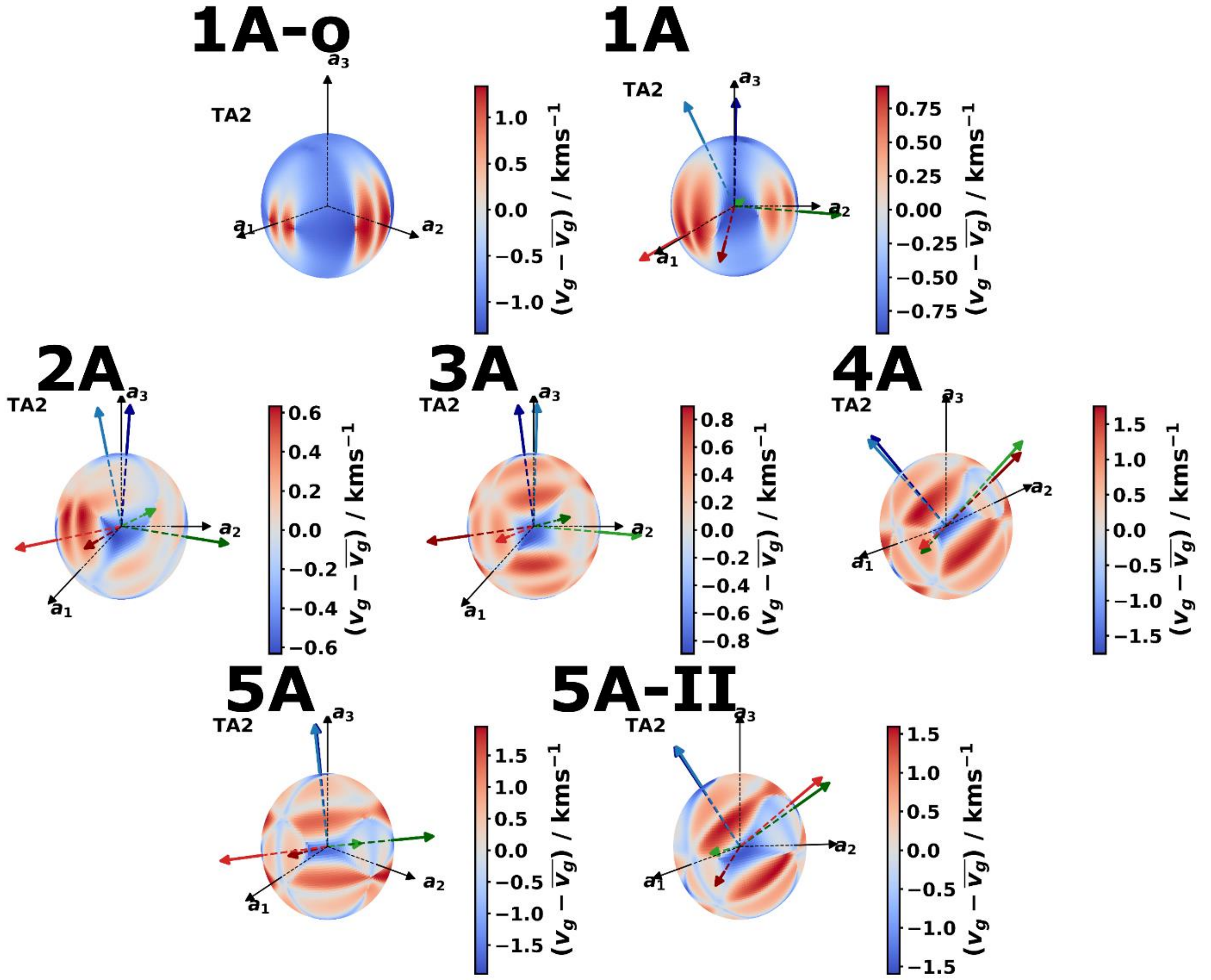

Figure S 19: Differences in the second (largely) transverse acoustic (TA_2) sound velocities and the corresponding mean values (see Table S 14) for all studied organic semiconductor crystals. The lattice vectors, $\boldsymbol{a}_1$, $\boldsymbol{a}_2$, and $\boldsymbol{a}_3$, are shown as black arrows. Additionally, the long molecular axes (blue arrows), the short molecular axes (red arrows) and the normal vectors to the π-planes (green arrows) of the two molecules per unit cell are indicated. The three arrows with a slightly darker shade belong to one molecule, while the lighter ones belong to the other. As 1A-o contains four molecules per unit cell, no additional molecular directions are shown to avoid perceptual confusion.

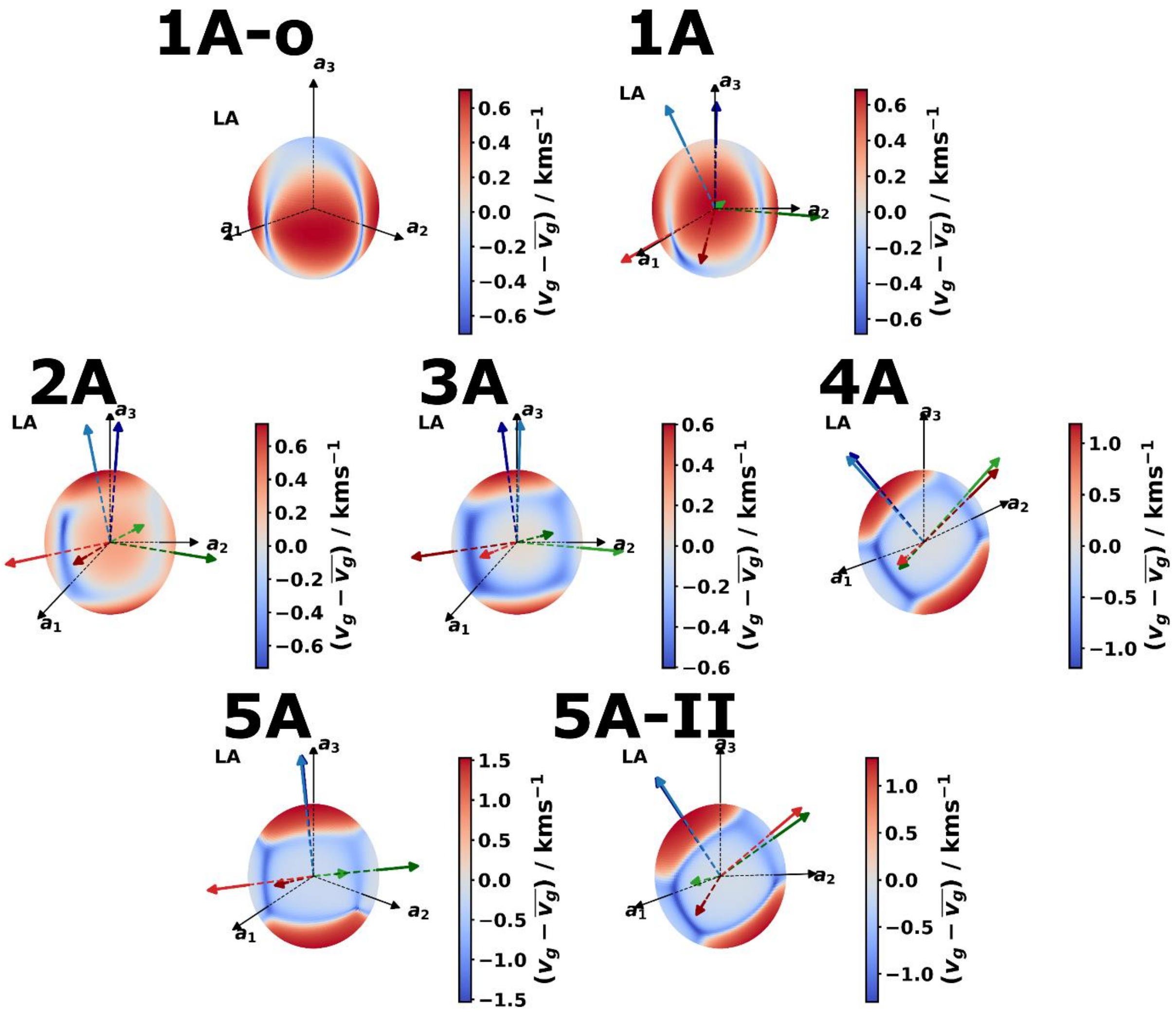

Figure S 20: Differences in the (largely) longitudinal acoustic (LA) sound velocities and the corresponding mean values (see Table S 14) for all studied organic semiconductor crystals. The lattice vectors, $\boldsymbol{a_1}$, $\boldsymbol{a_2}$, and $\boldsymbol{a_3}$, are shown as black arrows. Additionally, the long molecular axes (blue arrows), the short molecular axes (red arrows) and the normal vectors to the π-planes (green arrows) of the two molecules per unit cell are indicated. The three arrows with a slightly darker shade belong to one molecule, while the lighter ones belong to the other. As 1A-o contains four molecules per unit cell, no additional molecular directions are shown to avoid perceptual confusion.

S9. Effects of Polymorphism

Here, the most pronounced differences in the phonon-derived quantities introduced above and in the main text will be briefly discussed comparing monoclinic (1A) and orthorhombic benzene (1A-o) as well as the pentacene polymorphs I (5A) and II (5A-II).

S9.1 Orthorhombic benzene

As the orthorhombic phase of benzene (1A-o) contains twice the number of molecules per unit cell, it is impossible to assign the inter- and intramolecular modes on a one-to-one base as it was done for the remaining systems. However, one still can find interesting differences in many phonon-derived quantities discussed above and in the main text.

Regarding the phonon band structure (see Figure S 10), in orthorhombic benzene, the acoustic bands emerging from the centre of the first Brillouin zone, Γ, show almost perfectly linear dispersions over a comparably large frequency range like for the other systems. Moreover, their band widths are relatively similar for different directions in reciprocal space (amounting to ~1-2 THz). Only the band widths in the ΓZ direction show a slightly reduced dispersion: this direction corresponds to the lattice vector $\boldsymbol{a}_3$, along which the molecules pack less densely than in the other systems, reducing the effective stiffness of the system in that direction. With the aid of the PRs (i.e., by following the nearly linear band fragments with particularly high PRs), one can, however, observe that the actual band widths of the acoustic bands are even larger than suspected, since the bands frequently undergo avoided crossings (phonon hybridisations) involving other intermolecular bands belonging to the same irreducible symmetry representation (IRREP). This can, for example, be seen most clearly for the acoustic bands along ΓX and ΓZ in Figure S 10(b): at the first occasion at which they approach an optical band, phonon hybridisation occurs causing a small band gap to open (at ~1.6 THz and ~1.2 THz for the LA band). Depending on the hybridisation strength, this gap is smaller or larger, but in general, this phenomenon can be considered unfavourable for phonon transport[24–26] because of the (partial) loss of more strongly dispersing bands.

The *a priori* probability for avoided crossings is significantly reduced in the orthorhombic polymorph as the system exerts a space group with much more IRREPs.[27] Thus, it is less likely that two bands with the same symmetry meet, which is the prerequisite for phonon hybridisation. In order to emphasise this aspect, Figure S 21 shows the phonon band structure of 1A and 1A-o

along one exemplary path with the bands coloured according to their assigned IRREPs. It can be seen that the LA band in 1A meets two bands with the same IRREP along its path and, therefore, experiences two avoided crossings. Conversely, in 1A-o, the LA band only meets one band with the same IRREP along its way to Z so that only one avoided crossing is observed. Also the optical modes ≤ 4 THz (along this path) undergo drastically fewer avoided crossings although the density of bands is notably increased in this region in 1A-o.

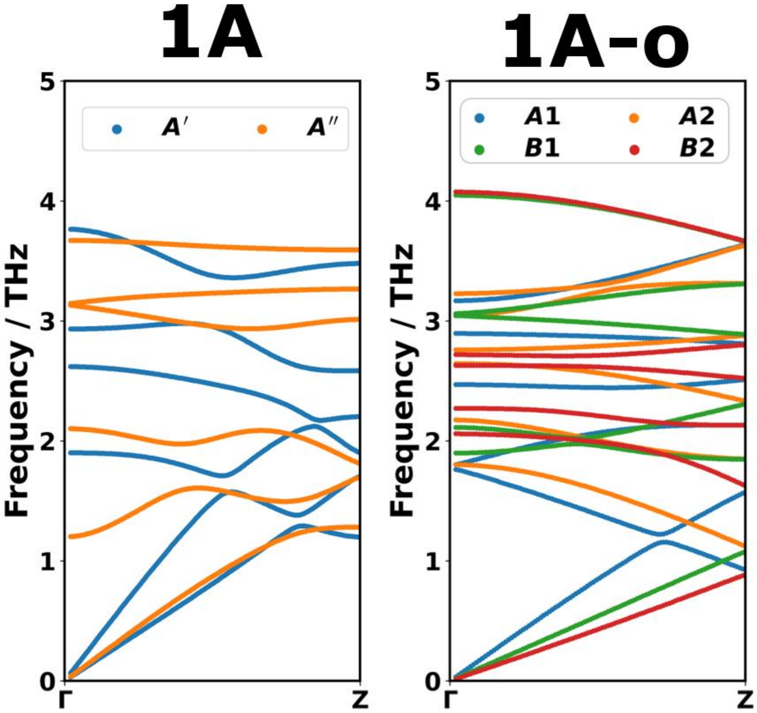

Figure S 21: Phonon band structure of monoclinic (1A) and orthorhombic (1A-o) benzene along the ΓZ path coloured according to the irreducible representations (IRREPs) of the symmetries of the eigenvectors. In the case of 1A, the group of the wave vector along this path is C_S (in Schoenflies notation) with two IRREPs (A', A''), while it is C_{2v} (in Schoenflies notation) for 1A-o with four IRREPs (A1, A2, B1, B2).

Besides the reduced probability of avoided crossings, the band structure of the orthorhombic polymorph with four molecules per unit cell shows more intermolecular modes (24=6×Z), which are, however, still well-separated from the higher intramolecular ones. Interestingly (see also the low-frequency DOS in Figure S 6), the additional intermolecular modes accumulate especially in the range ~2 – 3 THz, resulting in region of notably enhanced DOS compared to 1A. For higher, intramolecular modes, the phonon DOS of 1A and 1A-o look essentially identical.

Since these additional bands in 1A-o compared to 1A are predominantly rather flat, it is not surprising that a direct comparison of the densities of states per group velocity (DOGVs) considering only the low-frequency modes reveals an increased DOGV at lower group velocities in 1A-o (even when the DOGV is normalised by $3N$ in order to account for the different number of bands in both systems). However, also the DOGV at around 1.6-1.8 kms^{-1} in 1A-o is somewhat increased showing that there are also additional stronger-dispersing bands in 1A-o. These are likely to be the bands at ~4 THz at Γ, which correspond to RRIMMs with different phase shifts between neighbouring molecules.

Regarding the sound velocities, the average values of the different modes in 1A-o are consistently slightly higher than in 1A (and even somewhat higher than in 2A), but, in general, follow the same trends as 1A. Interestingly, the standard deviations for the sound velocities of the TA bands are significantly larger than in any of the monoclinic systems (1A, 2A, and 3A). This can also be seen clearly in the distributions of sound velocities in Figure S 18 and Figure S 19: the regions around the lattice vectors $\boldsymbol{a_1}$ and $\boldsymbol{a_2}$ show significantly higher group velocities, while exactly in the $\boldsymbol{a_1}$- and $\boldsymbol{a_2}$-directions the values are rather moderate. Interestingly, the LA sound velocity is largest at about 45° between $\boldsymbol{a_1}$ and $\boldsymbol{a_2}$. Looking at the crystal structure in the ($\boldsymbol{a_1}$ + $\boldsymbol{a_2}$)-direction, one notices that half of the molecules lie (roughly) in the same plane as the ($\boldsymbol{a_1}$ + $\boldsymbol{a_2}$)-vector (and in planes parallel to that), while the other half lies in planes approximately orthogonal to the former. This perfect face-to-edge herringbone packing of the molecules (meeting at nearly 90°) in this system seems to be especially suitable to allow a fast propagation of compression waves in this direction.

S9.2 Pentacene polymorph II

The direct comparison is much easier for the two considered polymorphs of pentacene. As the polymorph II (5A-II) shows certain differences in the packing arrangement (see Section S2), especially the intermolecular low-frequency phonons are expected to show some differences to the polymorph I (5A). As far as low-frequency intramolecular bands are concerned, Figure S 22 shows that their frequencies stay essentially the same. The largest for these modes is on the order of 0.3 THz (~4.05 THz in 5A and 3.75 THz in 5A-II) and is observed for the in-phase second-order bending modes, while all the differences for the remaining modes are smaller by at least a factor of ~3. This is most probably a result of the fact that in this mode in 5A, one of the two molecules

shows a strong rotational character in addition to the second-order bending due to hybridisation with a closely lying long axis RRIMM.

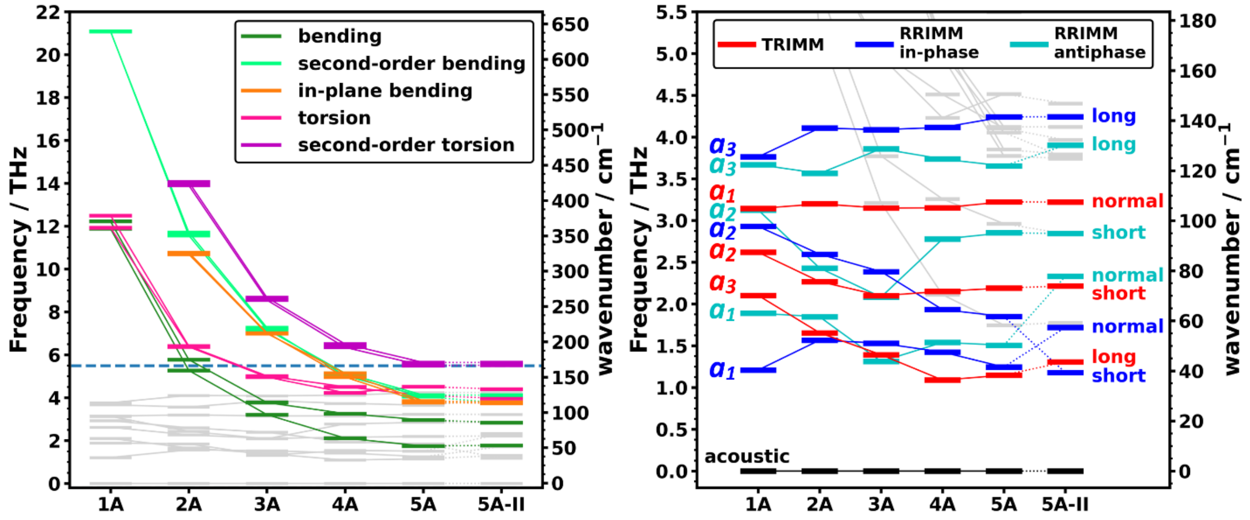

Figure S 22: Evolution of selected intramolecular and intermolecular frequencies at Γ, respectively, as a function of the molecular length from benzene (1A) to pentacene (5A) including the corresponding modes for pentacene polymorph II (5A-II). The frequencies are displayed as short horizontal bars, with the connecting lines serving as guides to the eye. In both panels, the frequencies belonging to the respective interesting regime (intra- or intermolecular) are shown in colour, while the other regime is shown as grey lines in the background. The narrow frequency interval shown in (e) is emphasised by the horizontal dashed line in (d). The type of the translational rigid intermolecular modes (TRIMMs) and rotational rigid intermolecular modes (RRIMs) are explicitly labelled by the lattice vectors $\boldsymbol{a_i}$ *on the left side, along which the translational motion occurs and by the molecular axes (long axis, short axis and the axis normal to both) on the right side, around which the rotation (roughly) is observed*

As expected, the changes of the intermolecular modes' frequencies are notably larger, where they amount to up to 0.83 THz for the difference in antiphase RRIMM around the π-plane normal. Also the in-phase RRIMM around the short axis experiences a significant drop in frequency, which is so large in magnitude (~-0.67 THz) that it comes to lie below the $\boldsymbol{a_3}$/long TRIMM. We attribute this to the much larger geometric offset between two layers of molecule, Δ_{short}, in 5A-II (see Table S 3). As a result, the molecules have more space to rotate around the short axis and, thus, the interaction strength (effective force constant) must be smaller, resulting in a lower frequency.

Interestingly, the TRIMMs are much less by the different polymorphic packing than the RRIMMs: the two higher ones ($\boldsymbol{a_1}$/normal and $\boldsymbol{a_2}$/short) essentially keep their frequency, while the frequency of the $\boldsymbol{a_3}$/long TRIMM increases by ~0.16 THz. Analysing the animations, this seems

to be the consequence of the increased bending character that the $\boldsymbol{a_3}$/long TRIMM in 5A additionally exhibits compared to the equivalent TRIMM in 5A-II.

Regarding the dispersion of the bands in both polymorphs, the low-frequency band structures (see Figure S 14 and Figure S 15) show many similarities. Even the band widths are typically nearly identical. One of the most pronounced differences apart from the slightly changed order of low-frequency intermolecular bands is the increased band width of the TA modes in 5A-II in ΓX and ΓY compared to 5A. The larger band widths can also be seen in the low-frequency zoom of the DOS in Figure S 6: here the typical quadratic (Debye-like) onset of the DOS (as a result of the linear dispersion of the acoustic bands) increases slightly more gradually in 5A-II. Apart from that, hardly any (significant) differences in the low-frequency DOS can be perceived, let alone in the higher intramolecular region > 7 THz. As a result, thermodynamic properties at elevated temperatures, for which also intramolecular modes contribute to a significant extent, are expected to exhibit nearly no differences between the two polymorphs (such as the heat capacities discussed in Section S10).

Also regarding the sound velocities, 5A and 5A-II are very similar. The average LA and TA_2 sound velocities are essentially identical with even the standard deviations being nearly the same. A more pronounced difference is observed in the TA_1 sound velocities: both the average TA_1 sound velocity and the associated standard deviation are slightly higher in 5A than in 5A-II, although the distributions of group velocity differences look practically identical for 5A and 5A-II (see Figure S 18 to Figure S 20).

Concerning the group velocities of the optical modes, the maximum of the low-frequency DOGV is slightly shifted to lower values, implying that among the bands with frequencies ≤ 7 THz, the group velocities are somewhat reduced in 5A-II compared to 5A.

S10. Molar heat capacities

It turns out that at 300 K, all the studied systems display nearly the same value of the normalised heat capacity, $C_V/(3Nk_B)$, (~0.3324) as discussed in the main text. Based on this observation, one can crudely estimate the molar heat capacity, which is typically the one measured in experiments, in the following way:

$$c_V^m(300\ K) = \left(\frac{C_V}{3Nk_B}\right)\Big|_{300\ K} \frac{3N}{Z}\, k_B N_A = 0.3324\, \frac{3N}{Z}\, k_B N_A \approx \frac{N}{Z} \cdot R \qquad \text{(S18)}$$

Here, N (Z) is the number of atoms (molecules) per unit cell and k_B, N_A, and R are the Boltzmann constant, the Avogadro constant, and the universal gas constant, respectively. This estimation shows that as a result from all systems showing a very similar value of $C_V/(3Nk_B)$ at 300 K, the molar heat capacity at room temperature is solely a function of the number of atoms per molecule, N/Z. In fact, the proposed estimation above relying only on this single variable provides a more than satisfactory agreement with experimental heat capacity data, although those were measured at constant pressure rather than at constant volume (C_p instead of C_V). The experimental values of the molar heat capacity (at constant pressure) for benzene[28,29], naphthalene[29,30], anthracene[29,31], tetracene[32], and pentacene[32] are shown as open circles in Figure S 23. Note that the experimental values are always slightly underestimated. One reason for this is that, in general, the relation $C_p > C_V$ holds at finite temperatures, since C_V implicitly neglects the influence of thermal expansion.[33]

In addition to the aspects already discussed in the main text it can be seen that polymorphism does not seem to have a notable effect on the heat capacity at constant volume. The intramolecular modes, which increasingly gain importance at higher temperatures, are too similar so that they obscure the pronounced but comparably few differences in the low-frequency region. Only at very low temperatures (see Figure S 23(c)), one can see that $C_V/(3Nk_B)$ is slightly smaller for 1A-o than for 1A. This observation emphasises the findings discussed in Section S9.1: the weight of the low-frequency (intermolecular) DOS is slightly shifted to higher frequencies. Therefore, somewhat higher temperatures are needed to thermally activate those phonons' contribution to the heat capacity.

Conversely, the (normalised) heat capacity of the 5A and 5A-II does not even show significant differences in this low-temperature region. Obviously, the few shifts in frequencies and the few differences in band dispersion are too minor to achieve notable variations in $C_V/(3Nk_B)$.

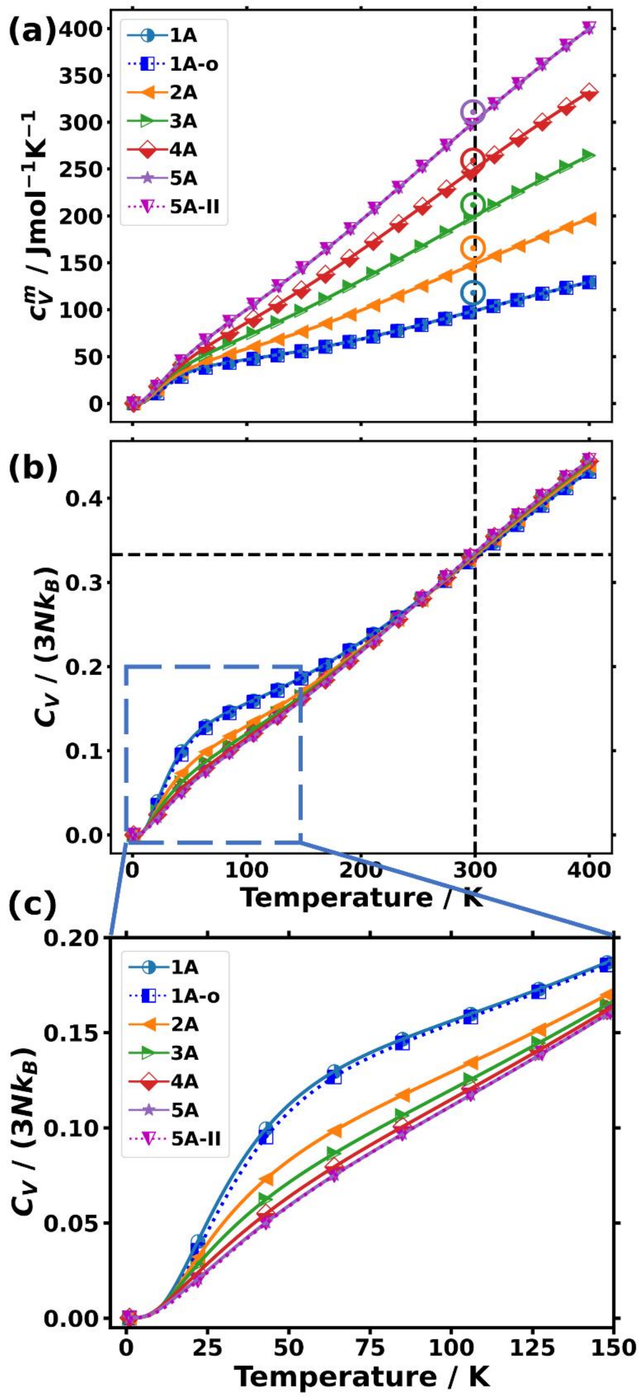

Figure S 23: (a) Molar heat capacity and (b) heat capacity per unit cell normalised with the classical Dulong-Petit limit (3Nk$_B$) as a function of temperature for the studied organic semiconductor crystals. The vertical dashed lines in (b) indicate the temperature of 300 K, while the horizontal one emphasises the common value in the normalised heat capacities of ~0.3324. The open circles in (a) indicate the experimentally determined values for the molar constant-pressure heat capacity for benzene[28,29], naphthalene[29,30], anthracene[29,31], tetracene[32], and pentacene[32]in the corresponding colours. (c) Zoom into the region indicated by the dashed box. Note that the symbols on top of the lines do not correspond to the actually calculated data points (those lie much more densely), but rather serve as guides to the eye.

S11. Comparison of low-frequency intramolecular between the crystals and the isolated molecules

Interesting insight into the non-covalent interactions due to the molecular packing in the studied organic crystals can be obtained by comparing the frequencies of eigenmodes in the isolated molecules to the equivalent Γ-phonons in the crystalline systems. The molecular frequencies in the isolated molecules were calculated using the *Gaussian 16* package[34] (Rev. A.03) employing the same functional and van der Waals correction as used in the periodic calculations (see Section S4.1) with a 6-311++G(d,p) basis set. These calculations are publicly available in the same dataset[1] as the periodic calculations in the NOMAD database.

Figure S 24 shows that all frequencies of the molecular vibrations experience a shift to higher frequencies when the molecule packs in the crystals. This is to be expected as the presence of the other molecules in the crystalline environment additionally exerts intermolecular, non-covalent interactions on the molecule such that the effective stiffness increases. Notably, the shifts to higher frequency in the crystals are more pronounced for longer the molecules: the average frequency shift in pentacene equals 1.30 THz (5A) and 1.23 THz (5A-II), decreases to 1.02 THz for tetracene, 0.80 THz for anthracene, 0.44 THz for naphthalene, and 0.19 THz for benzene. This reflects the stronger intermolecular interactions present in the crystals built of the longer acenes.

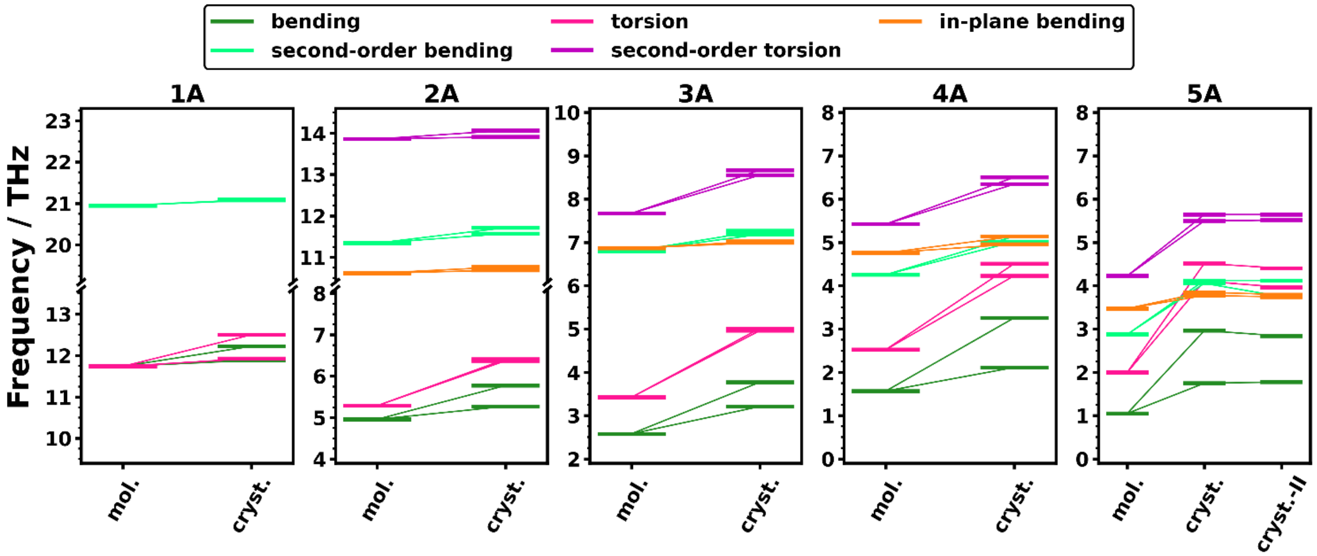

Figure S 24: Comparison of the frequencies selected intramolecular modes between the isolated molecular systems ("mol.") and the crystalline systems ("cryst."). In the case of pentacene, also the frequencies in the polymorph 5A-II ("cryst.-II") are shown. Note that every panel covers the same absolute frequency range of 8 THz.

REFERENCES

1 T. Kamencek and E. Zojer, 2021. NOMAD Dataset Phonons Crystalline Acenes, DOI: https://dx.doi.org/10.17172/NOMAD/2021.09.28-1

2 C. R. Groom, I. J. Bruno, M. P. Lightfoot and S. C. Ward, *Acta Crystallogr. Sect. B Struct. Sci. Cryst. Eng. Mater.*, 2016, **72**, 171–179.

3 G. E. Bacon, N. A. Curry, S. A. Wilson and R. Spence, *Proc. R. Soc. London. Ser. A. Math. Phys. Sci.*, 1964, **279**, 98–110.

4 G. J. Piermarini, A. D. Mighell, C. E. Weir and S. Block, *Science.*, 1969, **165**, 1250–1255.

5 S. C. Capelli, A. Albinati, S. A. Mason and B. T. M. M. Willis, *J. Phys. Chem. A*, 2006, **110**, 11695–11703.

6 C. P. Brock and J. D. Dunitz, *Acta Crystallogr. Sect. B*, 1990, **46**, 795–806.

7 D. Holmes, S. Kumaraswamy, A. J. Matzger and K. P. C. Vollhardt, *Chem. - A Eur. J.*, 1999, **5**, 3399–3412.

8 R. B. Campbell, J. M. Robertson and J. Trotter, *Acta Crystallogr.*, 1962, **15**, 289–290.

9 C. C. Mattheus, A. B. Dros, J. Baas, A. Meetsma, J. L. De Boer and T. T. M. Palstra, *Acta Crystallogr. Sect. C Cryst. Struct. Commun.*, 2001, **57**, 939–941.

10 K. Momma and F. Izumi, *J. Appl. Crystallogr.*, 2011, **44**, 1272–1276.

11 G. Kresse, M. Marsman and J. Furthmüller, VASP the Guide, https://cms.mpi.univie.ac.at/vasp/vasp.pdf, (accessed 3 April 2019).

12 S. Grimme, J. Antony, S. Ehrlich and H. Krieg, *J. Chem. Phys.*, 2010, **132**, 154104.

13 S. Grimme, S. Ehrlich and L. Goerigk, *J. Comput. Chem.*, 2011, **32**, 1456–1465.

14 T. Kamencek, S. Wieser, H. Kojima, N. Bedoya-Martínez, J. P. Dürholt, R. Schmid and E. Zojer, *J. Chem. Theory Comput.*, 2020, **16**, 2716–2735.

15 G. P. Francis and M. C. Payne, *J. Phys. Condens. Matter*, 1990, **2**, 4395–4404.

16 P. Vinet, J. R. Smith, J. Ferrante and J. H. Rose, *Phys. Rev. B*, 1987, **35**, 1945–1953.

17 A. Togo and I. Tanaka, *Scr. Mater.*, 2015, **108**, 1–5.

18 R. J. Bell, P. Dean and D. C. Hibbins-Butler, *J. Phys. C Solid State Phys.*, 1970, **3**, 2111–2118.

19 J. T. Edwards and D. J. Thouless, *J. Phys. C Solid State Phys.*, 1972, **5**, 807–820.

20 J. Canisius and J. L. van Hemmen, *J. Phys. C Solid State Phys.*, 1985, **18**, 4873–4884.

21 T. Kamencek, N. Bedoya-Martínez and E. Zojer, *Phys. Rev. Mater.*, 2019, **3**, 116003.

22 A. Stukowski, *Model. Simul. Mater. Sci. Eng.*, , DOI:10.1088/0965-0393/18/1/015012.

23 C. F. Beards, in *Structural Vibration: Analysis and Damping*, ed. C. F. B. T.-S. V. Beards, Butterworth-Heinemann, Oxford, 1996, pp. 129–156.

24 M. Christensen, A. B. Abrahamsen, N. B. Christensen, F. Juranyi, N. H. Andersen, K. Lefmann, J. Andreasson, C. R. H. H. Bahl and B. B. Iversen, *Nat. Mater.*, 2008, **7**, 811–815.

25 W. Li, J. Carrete, G. K. H. H. Madsen and N. Mingo, *Phys. Rev. B*, 2016, **93**, 1–5.

26 T. Zhu, K. Swaminathan-Gopalan, K. J. Cruse, K. Stephani and E. Ertekin, *Adv. Funct. Mater.*, 2018, **28**, 1–8.

27 S. F. A. Kettle, *Symmetry and Structure: Readable Group Theory for Chemists*, Wiley, 3rd edn., 2008.

28 R. G. Ross, P. Andersson and G. Bäckström, *Mol. Phys.*, 1979, **38**, 377–385.

29 R. G. Ross, P. Andersson and G. Bäckström, *Mol. Phys.*, 1979, **38**, 527–533.

30 J. P. McCullough, H. L. Finke, J. F. Messerly, S. S. Todd, T. C. Kincheloe and G. Waddington, *J. Phys. Chem.*, 1957, **61**, 1105–1116.

31 M. Radomska and R. Radomski, *Thermochim. Acta*, 1980, **40**, 405–414.

32 M. Fulem, V. Laštovka, M. Straka, K. Růžička and J. M. Shaw, *J. Chem. Eng. Data*, 2008, **53**, 2175–2181.

33 D. C. Wallace and H. Callen, *Am. J. Phys.*, 2005, 40, 1718–1719.

34 M. J. Frisch, G. W. Trucks, H. B. Schlegel, G. E. Scuseria, M. A. Robb, J. R. Cheeseman, G.

Scalmani, V. Barone, G. A. Petersson, H. Nakatsuji, X. Li, M. Caricato, A. V Marenich, J. Bloino, B. G. Janesko, R. Gomperts, B. Mennucci, H. P. Hratchian, J. V Ortiz, A. F. Izmaylov, J. L. Sonnenberg, D. Williams-Young, F. Ding, F. Lipparini, F. Egidi, J. Goings, B. Peng, A. Petrone, T. Henderson, D. Ranasinghe, V. G. Zakrzewski, J. Gao, N. Rega, G. Zheng, W. Liang, M. Hada, M. Ehara, K. Toyota, R. Fukuda, J. Hasegawa, M. Ishida, T. Nakajima, Y. Honda, O. Kitao, H. Nakai, T. Vreven, K. Throssell, J. A. Montgomery Jr., J. E. Peralta, F. Ogliaro, M. J. Bearpark, J. J. Heyd, E. N. Brothers, K. N. Kudin, V. N. Staroverov, T. A. Keith, R. Kobayashi, J. Normand, K. Raghavachari, A. P. Rendell, J. C. Burant, S. S. Iyengar, J. Tomasi, M. Cossi, J. M. Millam, M. Klene, C. Adamo, R. Cammi, J. W. Ochterski, R. L. Martin, K. Morokuma, O. Farkas, J. B. Foresman and D. J. Fox, *Inc., Wallingford CT*, 2016.
